# Hydro-geomorphological drivers across scales shape the trajectory of coastal wetland restoration

**DOI:** 10.1038/s41467-026-71992-x

**Published:** 2026-04-22

**Authors:** Junlin Ren, Sikai Wang, Tingting Zhang, Jun Ma, Ting Zhang, Chuyu Cheng, Kang Zhang, Changlin Xu, Johan van de Koppel, Daphne van der Wal, Tjeerd J. Bouma, Fangyan Cheng, Ping Zhuang, Feng Zhao

**Affiliations:** 1 East China Sea Fisheries Research Institute, Chinese Academy of Fishery Sciences, Shanghai, China; 2 College of Marine Living Resource Sciences and Management, Shanghai Ocean University, Shanghai, China; 3 Key Laboratory of East China Sea Fishery Resources Exploitation, Ministry of Agriculture and Rural Affairs, Shanghai, China; 4 Shanghai Engineering Technology Research Center for Fisheries Resource Enhancement and Ecological Restoration in the Yangtze River Estuary, Shanghai, China; 5 School of Life Sciences, Fudan University, Shanghai, China; 6 Key Laboratory of Marine Ecological Monitoring and Restoration Technologies, Ministry of Natural Resources, Shanghai, China; 7 East China Sea Ecology Center, Ministry of Natural Resources, Shanghai, China; 8 School of Mathematical Sciences, Shanghai Jiao Tong University, Shanghai, China; 9 State Key Laboratory for Vegetation Structure, Function and Construction (VegLab), Ministry of Education Key Laboratory for Earth Surface Processes, and College of Urban and Environmental Sciences, Peking University, Beijing, China; 10 Department of Estuarine and Delta Systems, Royal Netherlands Institute for Sea Research (NIOZ), Yerseke, The Netherlands; 11 Conservation Ecology Group, Groningen Institute for Evolutionary Life Sciences, University of Groningen, Groningen, The Netherlands; 12 Faculty of Geo-Information Science and Earth Observation (ITC), University of Twente, Enschede, The Netherlands; 13 Faculty of Geosciences, Department of Physical Geography, Utrecht University, Utrecht, The Netherlands; 14 State Key Laboratory of Subtropical Silviculture, Zhejiang A & F University, Hangzhou, China

**Keywords:** Restoration ecology, Wetlands ecology

## Abstract

Coastal wetlands provide essential ecosystem services but continue to decline globally, driving demand for effective restoration. Managed realignment, which relocates sea defenses landward to reinstate tidal exchange, is a key nature-based solution for creating self-sustaining wetlands. However, unpredictable restoration outcomes highlight the need for a framework to understand the underlying physical drivers and prioritize sites. Using four decades of satellite data from 69 global sites, we show that over 80% of the restoration projects maintained or expanded wetlands. These trajectories are primarily shaped by regional sediment supply, local tide-relative elevation, and internal tidal creek connectivity. Extending this framework globally, we estimate about 920 square kilometres of wetlands lost since the 1990s could be restored under current physical conditions. Recoverable areas in Asia, the Americas, and Europe exceed the 30% target of the Kunming-Montreal Global Biodiversity Framework. By linking outcomes to multi-scale physical contexts, our results provide a framework for prioritizing restoration and advancing global biodiversity and climate goals.

## Abstract (web summary)

A global study using 40 years of data from 69 sites shows wetland restoration succeeds when sediment, elevation and tidal creeks align across spatial scales. About 920 km² of lost wetlands worldwide are estimated to be potentially restorable.

## Introduction

Coastal wetlands are highly valuable ecosystems, providing essential services from biodiversity support to carbon sequestration^1,2^. However, these vital habitats are rapidly disappearing due to a combination of direct human impacts and climate change^3^. In response, the Kunming–Montreal Global Biodiversity Framework has set goals to restore 30% of degraded coastal ecosystems by 2030, underscoring the urgent need for effective and scalable restoration solutions.

To meet these goals, Nature-based Solutions (NbS) are increasingly promoted as strategies for climate adaptation and ecosystem restoration^4–6^. In coastal wetlands, NbS range from vegetative planting for living shorelines and hydrological reconnection of reclaimed lands to thin-layer sediment placement for marsh surface elevation and coastal resilience^7–10^. Specifically, managed realignment (MR), where existing sea defenses are moved inland to reinstate intertidal habitats, has emerged as a key NbS for restoring large-scale coastal wetland systems^11^. MR’s significance lies in its distinct ability to harness natural hydro-geomorphological processes to create long-term, self-sustaining habitats^7,12^. This approach offers a proactive and cost-effective strategy for climate adaptation, providing a globally relevant model for addressing both historical wetland loss and future sea-level rise by enabling dynamic ecosystems to naturally migrate inland. In this study, we focus on MR in non-mangrove tidal wetlands, including tidal flats and marshes.

Despite its immense potential and growing popularity among Nature-based Solutions, the overall footprint of managed realignment remains modest and unevenly documented across available inventories^13–15^. Furthermore, restoration success based on MR often varies widely among sites, influenced by physical processes (sediment supply, hydrology, and topography), restoration duration, and site configuration^9,16–18^. Consequently, the large-scale effectiveness of such efforts in restoring coastal wetland ecosystems remains unclear. This knowledge gap hinders our ability to assess whether restoration efforts are on track to meet the global restoration goals set for 2030. Numerous studies have separately highlighted the importance of individual hydro-geomorphological drivers such as regional sediment availability, site elevation, and tidal creek networks in shaping tidal flat expansion and tidal marsh colonization^19–21^, even though these factors are inherently interdependent. What remains missing is a unifying framework that integrates these multi-scale drivers to identify high-potential restoration areas and quantify the processes that determine restoration success.

Here, we hypothesized that hydro-geomorphological constraints operating across spatial scales would emerge as the dominant and most transferable drivers of coastal wetland restoration outcomes. Specifically, we expected that regional-scale sediment supply, site-scale relative elevation, and sub-site drainage structure would consistently explain variation in recovery trajectories across managed realignment (MR) sites. To test this hypothesis, we analyzed four decades of satellite imagery across 69 MR sites in Europe, North America and Oceania. We quantified spatiotemporal changes in tidal flat and marsh extent, evaluated each physical driver at its respective spatial scale, and then integrated these effects within a joint hierarchical model accounting for site heterogeneity. Building on these insights, we further extended our framework globally to identify restoration hotspots, illustrating the potential for MR to help achieve global biodiversity goals.

## Results

### Multi-decadal trajectories of restored coastal wetlands

**Fig. 1:**
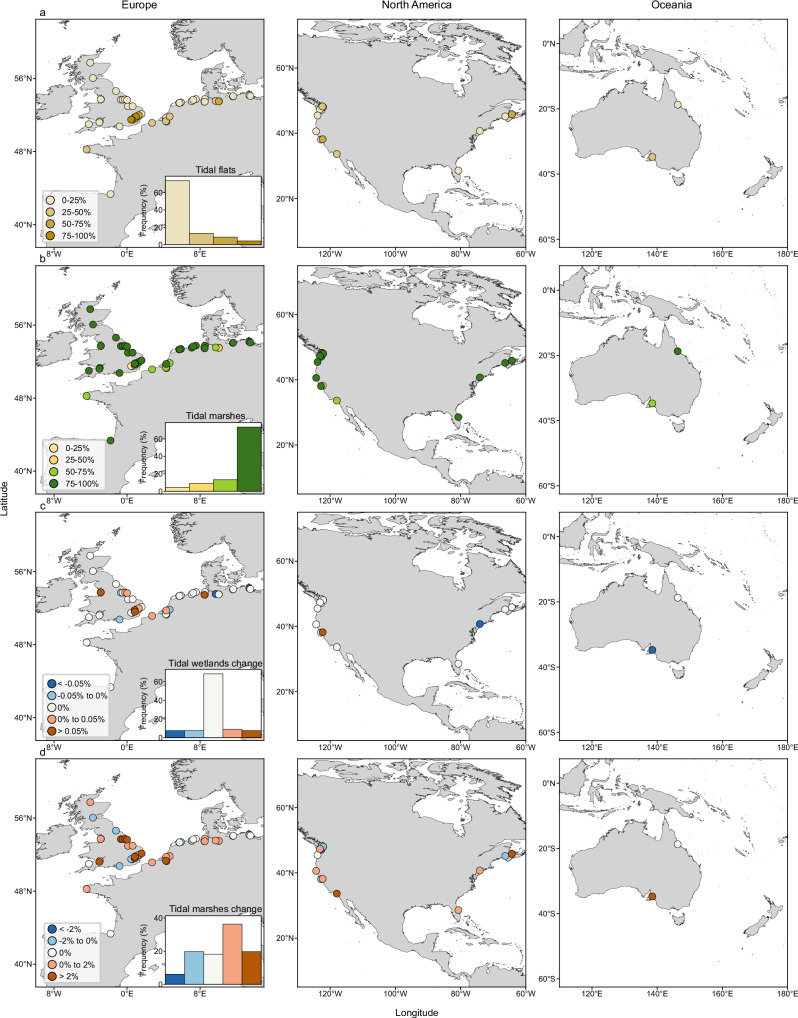
Multi-decadal changes in tidal wetlands across managed realignment sites in Europe, North America, and Oceania. Composition of tidal wetlands in the most recent mapped year, displayed as the percentage of area occupied by tidal flats (**a**) and tidal marshes (**b**). Annual change rates (% yr^−1^) for total tidal wetland extent (**c**) and tidal marsh composition (**d**). Insets show the corresponding across-site frequency distributions. Base maps were generated using public domain data from Natural Earth (https://www.naturalearthdata.com/).

Using four decades of satellite imagery, we assessed restoration success across 69 coastal wetland restoration sites spanning Europe, North America, and Oceania, and found that tidal wetlands (i.e., tidal flats and marshes) have been successfully maintained or expanded in most sites (Fig. 1c). Over 70% of these sites experienced marsh recovery (tidal marsh change rate, % yr^−1^ > 0) or have maintained full marsh cover since restoration began, leading to no detectable change (tidal marsh change rate = 0; Fig. 1d). Currently, most sites are dominated by marsh vegetation (marsh cover >75%, Fig. 1b), whereas a smaller subset remain predominantly tidal flats (Fig. 1a).

### Multi-scale hydro-geomorphological drivers of coastal wetland restoration success

**Fig. 2:**
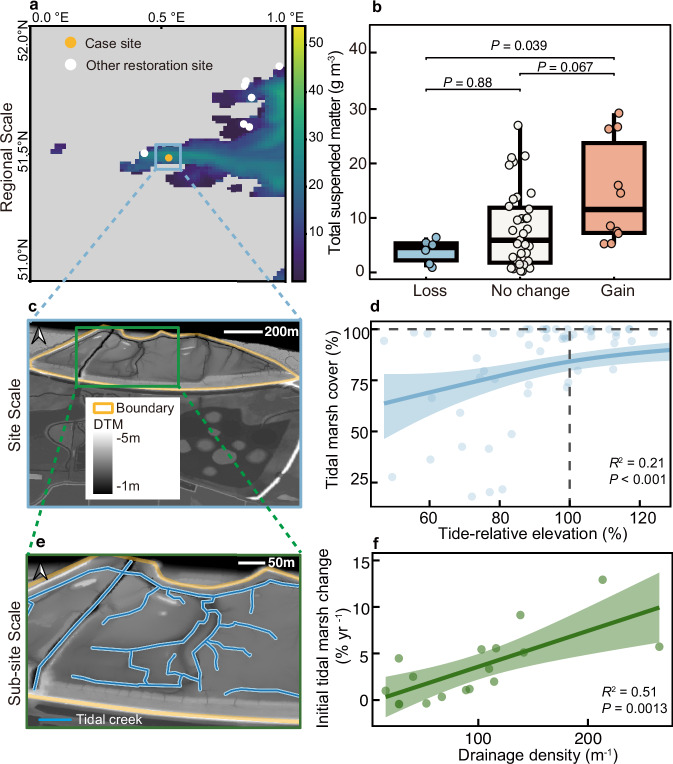
Multi-scale hydro-geomorphological drivers of tidal wetland restoration outcomes. Panels are organized by spatial scale: regional (**a**, **b**), site (**c**, **d**), and sub-site (**e**, **f**). **a** Regional distribution of total suspended matter (TSM). The focal site (Allfleets Marsh, UK) is highlighted. **b** TSM concentrations grouped by the annual change in tidal wetland extent (% yr^−1^): loss (<0), no change (=0), and gain (>0). For the boxplots, the center line indicates the median, box limits represent the upper and lower quartiles, and whiskers extend to 1.5 interquartile ranges, with individual points representing site-level observations; *P*-values indicate post hoc pairwise comparisons (Dunn’s test). **c** High-resolution elevation map (Digital Terrain Model, DTM) of the focal site. **d** Non-linear relationship between relative elevation and predicted tidal marsh cover. Relative elevation is defined as the percentage of the site’s mean elevation relative to the local tidal range (between mean low and high water spring tides). The solid line and shading represent the fitted trend and 95% confidence interval from a generalized additive model. **e** Tidal creek network within the focal site. **f** Linear relationship between early tidal marsh colonization rate and drainage density across restoration sites. Shaded areas represent 95% confidence intervals. This analysis is limited to sites with initial marsh cover <80% and relative elevation <100% to ensure measurable colonization periods. Base maps in **a** were generated using long-term average surface TSM data derived from the GlobColour archive (https://hermes.acri.fr/). Background elevation models in **c** and **e** were derived from the England 1 m Composit**e** DTM provided by the UK Environment Agency, available under the Open Government License (https://www.data.gov.uk/).

Multi-scale hydro-geomorphological drivers collectively shape the trajectory of tidal wetland restoration (Fig. 2). At the regional scale, previous studies across globally distributed estuaries have indicated that tidal wetland expansion or retreat is closely linked to sediment availability, along with tidal range. We assessed whether this relationship also holds true for restoration sites by comparing total suspended matter (TSM) concentrations across sites grouped according to their changes in tidal wetland extent (% yr^−1^): loss (<0, i.e., wetland area decreased), no change (=0, stable area), and gain (>0, area increased). Sites experiencing wetland expansion exhibited significantly higher TSM concentrations than sites with net losses (*P* = 0.039, Dunn’s post hoc test), and showed a similar tendency when compared with no-change sites (*P* = 0.067, Fig. 2a, b). Consistent results were obtained from the ordinal logistic regression, with higher TSM concentrations significantly increasing the odds that sites exhibit a more positive restoration trajectory (along the loss–stable–gain gradient, *P* = 0.0043), while tidal range showed no significant effect (*P* = 0.31; Supplementary Table 1).

At the site scale, elevation relative to the tidal range emerged as a crucial control of post-restoration marsh cover (Fig. 2c, d). To examine the correlates of variation in tidal marsh cover, we constructed a generalized additive model (GAM), which after model selection retained relative elevation as the sole statistically significant predictor (see “Methods”, *R*² = 0.21, *P* < 0.001, Fig. 2d). Relative elevation showed a threshold-like relationship with vegetation recovery. Within the active tidal frame (relative elevation <100%), marsh cover generally increased with elevation, although a few sites maintained relatively high cover at lower positions. Above the active tidal frame (relative elevation >100%), where sites are only inundated during extreme high-water events, marsh cover approached saturation (Fig. 2d). Notably, approximately one-third of the restoration sites exhibited a mean relative elevation below 80% (Supplementary Fig. 1). These low-lying sites drove the majority of limited vegetation recovery cases, ultimately remaining as persistent tidal flats.

At the sub-site scale, the efficiency of tidal creek networks facilitated early marsh colonization (Fig. 2e, f). Building on evidence that tidal creeks can promote early marsh establishment by enhancing drainage and propagule/sediment exchange, we tested whether variation in tidal creek network metrics helps explain differences in early colonization rates across restoration sites. Among sites within the active tidal frame (mean relative elevation <100%) with room for vegetation expansion (initial marsh cover <80%), early colonization rates were significantly positively correlated with drainage density (*R*² = 0.51, *P* = 0.0013, Fig. 2f). Conversely, sites characterized by longer mean unchanneled path lengths (UPL), indicative of less-developed tidal creek networks, exhibited slower colonization rates (*R*² = 0.21, *P* = 0.063, Supplementary Fig. 2).

Our findings indicated that different aspects of recovery were governed by specific physical controls across spatial scales. The joint hierarchical model successfully reproduced these scale-aligned relationships (Supplementary Table 2). Notably, wetland trajectories, marsh cover, and early colonization did not consistently improve together across sites (Supplementary Table 3), suggesting multiple, partially independent bottlenecks in managed realignment trajectories. These scale-aligned patterns remained robust even after incorporating additional hydro-geomorphological covariates (Supplementary Note 1 and Supplementary Table 4).

### Global potential for tidal wetland restoration

Building on these insights into the multi-scale hydro-geomorphological drivers of restoration success, we applied the same framework in a simplified, threshold-based manner to a global dataset of historical tidal wetland losses. This coarse screening allowed us to highlight regions where managed realignment (MR) is most likely to succeed under current sediment and elevation conditions.

**Fig. 3:**
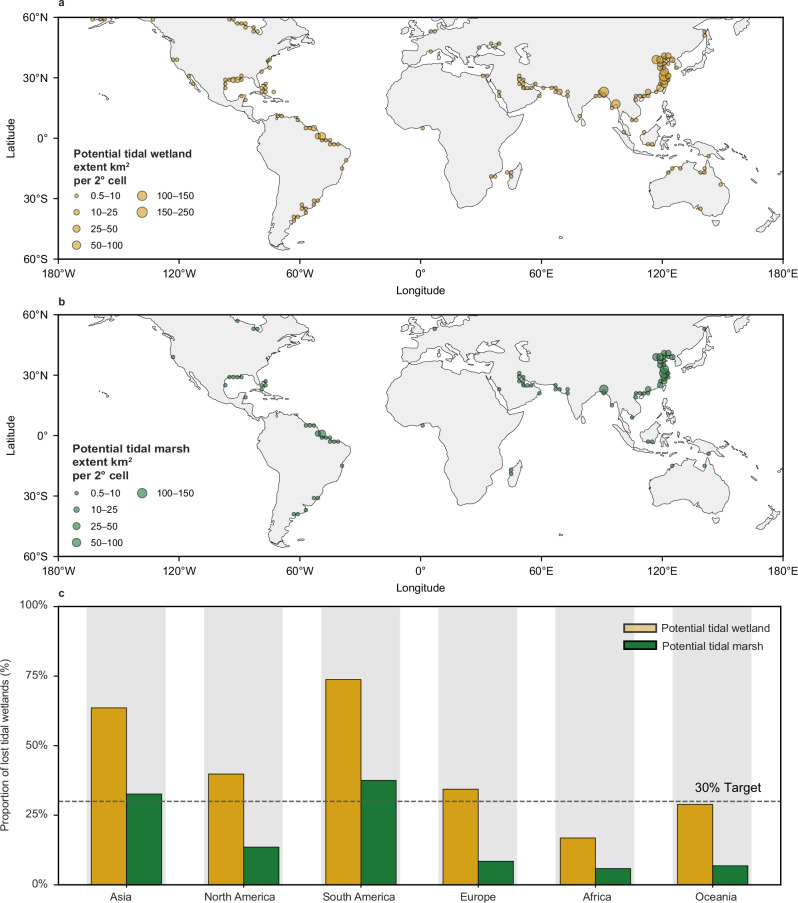
Global distribution of potential tidal wetland restoration opportunities. **a** Potential restoration extent for tidal wetlands, identified from historical (1999–2019) anthropogenically driven losses and screened for sufficient total suspended matter (TSM > 14.53 g m^−3^) under the optimistic sensitivity scenario. **b** Potential restoration extent for vegetated tidal marshes. This represents a subset of panel **a** restricted to regions where relative elevation exceeds approximately 70% of the local tidal range (between mean low and high water spring tides), representing the optimistic threshold for achieving high vegetation cover (see “Methods”). **c** Proportion of historically lost tidal wetlands meeting criteria for potential restoration to tidal wetlands and vegetated tidal marshes, summarized by continent. The dashed line indicates the 30% restoration target established by the Kunming–Montreal Global Biodiversity Framework. Base maps were generated using public domain data from Natural Earth (https://www.naturalearthdata.com/).

Globally, we identified ~924 km² of tidal wetlands lost to direct anthropogenic activities between 1999 and 2019 as potentially restorable through MR. Sensitivity tests with alternative sediment thresholds (TSM 14.53–26.84 g m^−3^, baseline = 20.37 g m^−3^) yielded estimates ranging from 568 to 1309 km². Of this area, about 389 km² (range 210–639 km² across elevation thresholds of 70–101%, baseline = 88%) meet the higher relative elevation thresholds for vegetated tidal marsh recovery. Notably, the conservative 101% bound represents elevations above the normal tidal frame that can still support high-marsh vegetation due to rare inundation from Highest Astronomical Tide (HAT) or occasional storm events. The remaining 535 km² (range: 277–842 km²) are more likely to support tidal flats following restoration (Fig. 3 and Supplementary Table 5). Although total estimates varied under alternative threshold assumptions, the uncertainty at the grid-cell level remained moderate (Supplementary Fig. 3). Across all cells, the mean standard deviation for the sediment-screened restoration potential was 0.18 km² (24.7% of the mean cell-level estimate of 0.71 km²). For the vegetation-eligible restoration potential, the corresponding mean standard deviation was 0.09 km² (28.7% of the mean estimate of 0.31 km²).

Continental-scale mapping revealed strong geographic heterogeneity in restoration potential (Fig. 3). Asia and South America contained the largest shares of historically lost tidal wetlands that met the sediment threshold, and many of these areas also fell within suitable elevation ranges for marsh establishment, indicating substantial opportunities for restoring both tidal flats and marshes through managed realignment. Hotspots were concentrated along the coasts of East and South Asia, particularly in China (e.g., the Yangtze River Delta) and Bangladesh, as well as along the northern coast of South America (Fig. 3a, b). Under optimistic threshold assumptions, estimated restoration potential approached or exceeded the 30% Kunming–Montreal target in all regions except Africa (Fig. 3c), with upper estimates in Asia and South America reaching approximately 70% of tidal wetland losses over the past two decades.

## Discussion

With growing recognition of the ecological and socio-economic importance of coastal wetlands, an increasing number of nature-based restoration projects have been implemented globally in recent years^8,22^. In this study, we compiled and mapped the spatial distribution of tidal wetlands at 69 managed realignment (MR) sites over the past four decades across Europe, North America, and Oceania, and then evaluated their restoration trajectories. Our results demonstrate that most sites exhibit positive restoration trajectories, characterized by gains in either vegetated tidal marshes or unvegetated tidal flats. Crucially, within a unified cross-scale analytical framework, we show that the success of managed realignment is governed by hydro-geomorphological processes operating at distinct spatial scales. Specifically, at the regional scale, total suspended matter (TSM) concentrations control whether tidal wetlands are maintained or expand; at the local site scale, elevation relative to tidal range determines the extent of vegetated tidal marsh cover; and at the sub-site scale, the efficiency of tidal creek networks regulates the early colonization rate of tidal marsh vegetation.

Building on this unified cross-scale analytical framework, we extended it globally to identify tidal wetlands that were historically lost due to direct human activities but remain physically restorable under current hydro-geomorphological conditions. Our analysis reveals substantial restoration potential exceeding 30% of the total wetland loss in some regions, particularly in sediment-rich and appropriately elevated areas across Asia, the Americas, and Europe. By translating insights derived from managed realignment experience into a globally applicable, physically grounded screening approach, our findings offer transferable guidance for implementing nature-based restoration strategies across diverse coastal settings worldwide and for supporting global restoration goals under the Kunming–Montreal Biodiversity Framework.

### Synthesizing the multi-scale hydro-geomorphological drivers of restoration success

Our large-scale spatio-temporal analyses allowed us to identify key drivers operating at three distinct spatial scales that shape managed realignment restoration trajectories. To our knowledge, large-scale and long-term evaluations of coastal wetland restoration success using remote sensing approaches remain scarce, particularly regarding Nature-based Solutions (NbS). Previous smaller-scale studies have often assessed restoration success by measuring vegetation diversity, carbon storage, and habitat quality for fish and migratory birds^23–25^. While these measures capture important facets of restoration outcomes, the spatial extent and temporal trajectory of restored tidal wetlands represent the most fundamental and integrative indicators of restoration performance.

Our results, derived from 69 coastal wetland restoration sites across Europe, North America, and Oceania, indicate that the majority of managed realignment projects yielded positive restoration outcomes. Specifically, tidal wetland extent (including both tidal flats and marshes) was maintained or expanded in over 80% of the sites. While around 80% of sites exhibited post-restoration increases in vegetated marsh area or achieved full marsh colonization shortly after restoration, a considerable number of sites or portions thereof remained as functioning tidal flats (Fig. 1). Although quantitative evidence linking restoration success to ecosystem service provision is more extensive for vegetated marshes than for unvegetated tidal flats, reflecting a historical research emphasis on plant-mediated functions^2,26^, tidal flats nonetheless provide key ecological functions, including shorebird foraging habitat, benthic food-web support, and contributions to sediment dynamics^27–29^. In this context, the persistence of tidal flats following managed realignment should be viewed not as a restoration failure, but as an alternative and potentially stable outcome, depending on local physical conditions and management objectives.

Our findings show that coastal wetland restoration sites exhibiting a reduction in tidal wetland extent generally have lower concentrations of total suspended matter (TSM), whereas regions where wetlands are expanding exhibit significantly higher TSM concentrations (Fig. 2b). This result aligns closely with observations at the global estuarine scale, which identify sediment availability as a primary constraint on wetland persistence and expansion^18^. Abundant sediment supply not only facilitates the expansion of restored wetlands but also promotes vertical accretion and elevation gain, accelerates both mineral and organic matter accumulation, and ultimately enhances vegetation growth^7,9,30^. In contrast, limited sediment availability can lead to wetland retreat and prevent sites from attaining the surface elevations needed to sustain normal ecological functions^31,32^. Notably, unlike some global-scale assessments that report a moderating role of tidal range, we did not detect an independent effect of tidal range on restoration outcomes in our managed realignment sites (see Supplementary Table 1), a pattern consistent with a global meta-analysis showing that the expansion or retreat of restored coastal wetlands is primarily governed by sediment availability rather than by tidal range or wetland elevation^9^.

In contrast to some previous studies^16^, we found at the local scale that neither project age (time since restoration) nor site size significantly influenced restoration success in terms of marsh cover. Instead, marsh cover was driven by relative elevation compared with the local tidal range, following a distinct nonlinear relationship (Fig. 2d and Supplementary Table 6). A plausible mechanism is that the lower elevation limit for marsh colonization is primarily constrained by inundation frequency^33^. When tidal disturbance falls below a critical threshold, marsh vegetation can rapidly expand across available habitat^34,35^.

Our analysis of relative elevation is based on the most recent high-resolution digital terrain models (DTMs), which were matched as closely as possible to contemporary marsh cover. Treating elevation as a site-specific, time-invariant driver simplifies the framework and facilitates cross-site comparison, although marsh platform elevations may continue to evolve following restoration through sediment accretion or erosion processes^16,36^. Despite this simplification, the observed nonlinear relationship highlights relative elevation as a robust site-scale constraint on marsh development following managed realignment.

At the sub-site scale, we found that in restoration sites characterized by frequent inundation and where vegetation colonization had not yet saturated during the early restoration period, greater tidal creek network efficiency significantly accelerated early marsh colonization rates (Fig. 2f and Supplementary Fig. 2). Tidal creek networks facilitate early-stage marsh development by enhancing drainage and moderating soil moisture, salinity, and redox conditions^37–40^. Where drainage density is low, persistently waterlogged surfaces and reduced redox potentials can restrict rapid plant establishment and stall successional progression, favoring only flood-tolerant pioneer species^35,41^. By contrast, well-developed creek networks promote more efficient substrate drainage and create localized ecological refugia that enhance seed germination and seedling survival, supporting higher plant diversity and denser vegetation cover, consistent with field and modelling studies of early marsh recruitment^42–44^.

Our results further demonstrate that, although sediment supply, relative elevation, and tidal creek networks are interdependent, they play distinct roles across spatial scales in shaping restoration trajectories (Supplementary Table 2). Embedding these drivers within a hierarchical cross-scale framework allows us to characterize their scale-dependent roles across spatial levels. In this framework, sediment availability functions at the regional scale as the fundamental building material, determining whether an area has the basic physical potential for tidal wetland recovery. Relative elevation acts at the site scale as the foundation, setting the topographic limits for successful marsh establishment once recovery is feasible. At the sub-site scale, tidal creek networks provide the internal plumbing system, regulating hydrological connectivity, drainage efficiency, and associated soil conditions that ultimately influence the pace and efficiency of vegetation colonization. By quantifying these scale-specific effects within a unified hierarchical model, our study complements existing qualitative and single-scale descriptions of restoration success by providing a quantitative, scale-explicit synthesis of the physical drivers shaping wetland restoration trajectories. This integrative perspective offers a practical basis for anticipating restoration outcomes, guiding the design of managed realignment interventions, and incorporating cross-scale physical processes into ecological restoration models.

### Towards optimized Nature-based restoration in coastal zones

Overall, our study provides critical insights for enhancing the design and management of Nature-based Solutions (NbS) in coastal wetland restoration. By elucidating how regional sediment supply, site elevation, and sub-site creek morphology jointly shape restoration trajectories, we underscore the importance of aligning restoration strategies with prevailing hydro-geomorphological conditions. To translate these insights into practice, sediment transport pathways and budgets should be considered early in project planning, and thin-layer sediment placement can be used to raise site elevation where vegetation establishment is desired^45,46^. In low-lying areas, constructing tidal creeks that mimic natural patterns at the start of restoration can further facilitate early vegetation colonization^38^. Together, these measures can help ensure more effective and resilient restoration outcomes across diverse coastal settings.

Beyond site-level design, we extended the framework globally to identify and quantify tidal wetlands that were historically lost due to direct anthropogenic activities, such as reclamation and hydrological modification, and that remain physically restorable under current sediment availability. Our analysis reveals that the potentially recoverable areas in regions such as Asia, the Americas, and Europe exceed the 30% restoration target set by the Kunming–Montreal Global Biodiversity Framework (Fig. 3). By prioritizing managed realignment (MR) implementation in areas that meet sediment supply thresholds, countries can strategically invest in Nature-based Solutions (NbS) to achieve more resilient and cost-effective outcomes. Furthermore, by integrating relative elevation data from existing restoration sites, we distinguished potential restoration trajectories into vegetated tidal marshes and unvegetated tidal flats. This differentiation enables tailoring of restoration goals to local ecological needs, whether focused on maximizing vegetation growth or providing critical foraging habitat for shorebirds^47^.

This screening-level, globally consistent approach is particularly valuable in data-poor regions (e.g., Africa), where long-term monitoring and comprehensive restoration inventories are often lacking, limiting the feasibility of data-intensive, site-specific models^48,49^. Because the proposed framework relies on a small number of physically interpretable variables that are globally available from remote sensing and modelling products, it provides a practical first-order screening tool for identifying physically plausible restoration opportunities globally. In such contexts, the framework is not intended to replace detailed local assessments, but rather to guide where limited resources for field surveys, pilot projects, and stakeholder engagement can be most effectively deployed.

To illustrate the broader applicability of our framework, we note that similar scale-dependent drivers underpin restoration success in other coastal ecosystems, even when ecosystem-specific mechanisms differ. Mangrove restoration is governed by biophysical and geomorphic processes that differ in important ways from those in temperate saltmarshes, including differences in sediment trapping and transport pathways, tidal-channel network properties, and vegetation–sediment feedbacks^50–52^. Even so, sediment availability remains a key constraint in many mangrove restoration contexts: TSM concentration has been shown to strongly influence the success of hydrological restoration by regulating sediment accretion and seedling establishment^9^. Still within mangrove systems, the micro-climate near tidal creeks can benefit the growth of planted mangroves^53^. Similar context dependence is also evident in coastal dunes, where the survival rate of transplanted American beachgrass increases with elevation, reflecting the importance of elevation-driven exposure and drainage conditions^54^. Together, these examples suggest that embedding multi-scale physical context into NbS design represents a unifying principle across coastal ecosystem types, even where restoration strategies and ecosystem structure differ.

### Uncertainties and interpretation of global restoration estimates

Overall, our global restoration estimates provide a conservative, process-based screening of physically plausible recovery space, but they inevitably carry uncertainty. By explicitly varying key sediment and elevation thresholds and considering limits to data coverage and representativeness, we clarify the robustness and transferability of the mapped patterns. Below, we quantify parameter sensitivity, discuss additional sources of uncertainty related to ecosystem type and dynamic elevation change, and provide guidance for interpreting regional differences in the resulting global maps.

To reflect parameter uncertainty within this parsimonious structure, we explored a range of suspended sediment thresholds (14.53–26.84 g m^−3^) and relative elevation thresholds (70–101% of the local tidal range), which together define a sensitivity envelope for the global estimates. Sediment requirements for wetland maintenance and recovery, however, are not universal and may vary with tidal range and hydrodynamic regime^18,55^. As a result, the framework may still overestimate restoration potential in macrotidal environments and underestimate it in microtidal systems, despite using relative elevation metrics. Moreover, even where sediment supply and elevation thresholds are met, local geomorphic and ecological factors, including wave exposure, substrate composition, salinity regimes, and propagule availability, may further constrain restoration feasibility and influence realized outcomes^7,56^.

Beyond parameter uncertainty, the transferability of this screening framework is constrained by data coverage and representativeness. While the number of managed realignment sites analysed here is larger than in most previous remote-sensing-based evaluations, it remains insufficient to support highly parameterized or fully process-based modelling at the global scale, particularly for sub-site processes where tidal creek analyses rely on a smaller subset of sites. Accordingly, we focus on a parsimonious, screening-level framework that emphasizes physically interpretable constraints. Empirically, the framework is informed primarily by managed realignment sites in Europe, with fewer sites from North America and Oceania, and therefore is most representative of temperate, minerogenic saltmarsh systems where elevation change is strongly influenced by external mineral sediment supply (Fig. 1). Transferability to organogenic marshes, where in situ organic production and biogenic feedbacks can play a larger role and creek-network properties may differ, may therefore be limited^56^. Accordingly, thresholds and resilience patterns inferred largely from minerogenic settings should not be directly extrapolated to organogenic marshes without additional local evidence.

In addition, wetland elevation is not static following managed realignment. Restored platforms may continue to accrete or erode through time, shifting relative elevation and altering the elevation window suitable for tidal marsh establishment^9,57,58^. These dynamic feedbacks between sediment supply, morphology, and vegetation are not explicitly resolved in the present screening framework, implying that long-term outcomes depend not only on initial sediment and elevation conditions but also on their subsequent evolution under local hydro-geomorphological processes.

The resulting global maps should be interpreted as a conservative, process-based screening of physically plausible recovery space rather than a comprehensive inventory of restoration pathways. Apparent low restoration potential can arise either because the mapped extent of direct anthropogenic tidal-wetland loss between 1999 and 2019 is small in a region, which limits the absolute area available for restoration within a loss-based framework^3^, or because only a smaller share of the mapped losses meets the physical thresholds used in the screening. In Oceania and Africa, mapped losses are relatively limited, and many coastlines are dominated by mangrove ecosystems that fall outside our non-mangrove focus (Fig. 3)^59–61^. In addition, many estuaries in these regions are wave-dominated, or mixed-energy settings with comparatively low to moderate suspended sediment availability^62,63^, so conservative sediment thresholds may underestimate physically plausible restoration space in these regions. In contrast, across Europe, the eastern USA, and Canada, mapped restoration potential is often limited mainly in absolute terms because direct anthropogenic losses during 1999–2019 are sparse in the underlying loss datasets (Fig. 3). Consequently, while our global estimates provide a crucial first-order screening, they cannot substitute for practical feasibility studies; these macro-scale projections must ultimately be complemented by site-specific assessments that consider local geomorphic context, ecosystem type, and management objectives.

Despite these uncertainties, as the global community advances efforts to restore 30% of degraded ecosystems by 2030 under the Kunming–Montreal Global Biodiversity Framework, our findings highlight the need to embed multi-scale physical drivers, such as sediment supply, elevation, and hydrological connectivity, at the heart of coastal restoration strategies. Rather than applying generalized solutions, effective Nature-based Solutions (NbS) must be tailored to these multi-scale contexts. Integrating these principles into the planning, design, and adaptive management of restoration initiatives can improve outcomes, enhance resilience, and enable more scalable interventions. More broadly, these insights support the development of evidence-based policies that align scientific understanding with practical implementation, positioning NbS as a cornerstone of global biodiversity conservation and climate adaptation.

## Methods

### The studied restoration sites

We compiled a global dataset of tidal wetland restoration sites by integrating three established restoration databases: the Online Managed Realignment Guide (OMReg) database^13^, the NOAA Restoration Atlas for the United States^15^, and the Database for Marine and Coastal Restoration Projects in Australia and New Zealand (ACRN)^14^. From these databases, we selected sites restored through managed realignment or functionally equivalent approaches, including deliberate dike breaching or removal aimed at restoring tidal exchange and facilitating natural wetland development (Supplementary Fig. 4).

To ensure consistency in restoration objectives and ecological processes, we restricted our analysis to projects targeting coastal wetlands, specifically unvegetated tidal flats and vegetated salt marshes. Projects primarily focused on mangrove restoration were excluded for two reasons. First, mangrove ecosystems are characterized by woody vegetation and distinct sedimentary, hydrodynamic, and creek-network feedbacks that differ fundamentally from those of tidal flats and salt marshes^50–52^. Second, mangrove-based managed realignment projects remain sparsely represented across the three databases, limiting their suitability for comparative analysis.

We further applied consistent spatial thresholds, boundary definitions, and data-processing procedures across all regions. Only sites larger than 10 hectares were included to ensure reliable detection of wetland change using 30-m resolution satellite imagery and to minimize habitat misclassification caused by mixed pixels. Site boundaries were delineated based on restoration design: for projects involving partial embankment removal, the original embankment footprint defined the site extent, whereas for projects with complete embankment removal, boundaries encompassed both the restored wetland and adjacent tidal waters to enable robust tracking of tidal wetland dynamics. In total, 69 restoration sites meeting these criteria were retained for analysis (Supplementary Table 7).

### Constructing the wetland dataset

Following the delineation of restoration site boundaries, we applied a mapping approach based on a decision-tree, unsupervised classification method that has been previously validated for tidal wetland mapping and is transferable across geographic regions (Supplementary Fig. 5)^64,65^.

For each restoration site, a time series of Landsat 5 TM, Landsat 7 ETM+, and Landsat 8 OLI imagery was compiled at three-year intervals from the year restoration began to 2024. To minimize the effects of cloud cover, snow/ice, and radiometric saturation, only high-quality pixels were retained in each period. Using these composite images, a rule-based classification approach was applied independently to each restoration site and time period. The algorithm sequentially identified and extracted three primary wetland classes based on spectral indices and their persistence. First, a pixel was classified as vegetated marsh if its Normalized Difference Vegetation Index (NDVI) exceeded 0.3 in more than 20% of the high-quality images within the three-year period. Second, for the remaining unclassified pixels, we calculated the Normalized Difference Water Index (NDWI). A pixel was classified as permanent water if its NDWI exceeded 0 in more than 85% of the images within the same period. Finally, the remaining intertidal pixels that met neither the persistent vegetation nor the permanent water criteria were classified as unvegetated tidal flats (Supplementary Fig. 5)^64^. As a result, each site was assigned a categorical wetland map for every three-year time step, enabling the reconstruction of wetland composition trajectories throughout the restoration period.

This classification approach has demonstrated high accuracy in European tidal wetlands and has proven consistent across Landsat 5 TM, Landsat 7 ETM+, and Landsat 8 OLI imagery^64,65^. To further assess its reliability in the selected restoration sites, validation was performed using high-resolution Google Earth images for 10 randomly selected sites during the vegetation season, representing a range of restoration ages. Because restoration started in different years across sites, the validation imagery was not constrained to a single calendar year. Instead, for each site, we selected a cloud-free Google Earth image acquired during the vegetation season in the year corresponding to the reported restoration age. For each restoration site, 50 validation points were randomly sampled and manually assigned to one of three classes (permanent water, vegetated marsh, or unvegetated flat), and then compared against the classification outputs. Overall accuracies exceeded 80%, and the corresponding Kappa coefficients were all above 0.76, confirming the robustness of the method for detecting changes in wetland composition over time (see details in Supplementary Table 8).

### Quantifying success in restoration sites

Based on the wetland maps generated at three-year intervals, we assessed restoration success at each site by tracking shifts in both vegetated and unvegetated wetland extents. Specifically, four measures were used to quantify restoration performance at each site: (i) the rate of total tidal wetland (vegetated tidal marsh and unvegetated tidal flat) expansion, (ii) the rate of vegetated tidal marsh expansion, (iii) the current tidal flat cover, and (iv) tidal marsh cover.

For each restoration site and time period, tidal wetland extent was calculated as a proportion of the site’s total area, while tidal marsh and tidal flat cover were calculated as proportions of the total tidal wetland area within the same period. A linear regression was fitted to each site’s time series to estimate long-term trends (% yr^−1^) in both tidal wetland extent and tidal marsh cover since the year restoration began. The most recent tidal flat and marsh cover were calculated as the proportion of the site occupied by tidal flats and vegetated marsh in 2022–2024. These four values were used to assess and compare recovery trajectories across sites. Histograms were generated to illustrate the frequency distribution of these measures across all sites.

### Linking hydro-geomorphology to restoration performance

To assess the influence of hydro-geomorphological drivers across three nested spatial scales, we first evaluated each scale independently by examining whether variation in regional-scale sediment supply, site-scale elevation, and sub-site drainage morphology was associated with differences in restoration outcomes and recovery dynamics among tidal wetland restoration sites, and then integrated these scale-specific effects within a joint hierarchical modelling framework to preserve scale-specific inference while synthesizing evidence across scales.

### Regional sediment availability and tidal wetland change

To evaluate the influence of regional sediment supply on restoration outcomes, we used total suspended matter (TSM) concentration as a proxy for long-term sediment availability. Both global-scale remote sensing analyses and site-level experimental studies have consistently demonstrated that the vertical accretion and lateral expansion of tidal marshes depend strongly on suspended sediment inputs^9,18^. High TSM levels support sediment deposition, facilitate elevation gain, and are therefore critical for the stability and growth of restored wetlands.

To test this hypothesis within the context of tidal wetland restoration, we examined whether variation in sediment supply corresponds with divergent wetland trajectories. Each restoration site with at least three time steps was categorized into one of three groups based on its long-term rate of tidal wetland extent change (% yr^−1^): loss (<0), no change (=0), and gain (>0). This grouping allowed for comparison of sediment availability across different restoration trajectories. Long-term average surface TSM estimates derived from MERIS imagery were obtained from the GlobColour archive (https://hermes.acri.fr/). The long-term mean TSM concentration was calculated within a buffer surrounding the restoration boundary to represent regional sediment supply, with buffer size matched to the spatial resolution of the TSM data (1 km for European sites and 4 km elsewhere).

Following the estimation of long-term sediment supply for each site, TSM concentrations were compared across the three wetland change categories using a Kruskal–Wallis test, followed by Dunn’s post hoc pairwise comparisons with Bonferroni-adjusted *P* values. To further quantify how sediment supply shifts the likelihood of sites transitioning along an ordered loss–stable–gain gradient, we fitted an ordinal logistic regression model with TSM as the primary predictor and tidal range as a covariate to account for hydrodynamic context, given prior evidence that tidal range may influence tidal wetland expansion under certain conditions^18,55^. We assessed the proportional odds assumption of the ordinal logistic regression using the Brant test, which indicated no significant deviation from the assumption (omnibus test statistic = 0.57, df = 2, *P* = 0.752). Finally, we extracted the model coefficients and corresponding odds ratios to evaluate the magnitude and direction of the effects of TSM on wetland change category (Supplementary Fig. 6 and Supplementary Table 1).

### Site elevation and tidal marsh coverage

The relative elevation of tidal wetlands, defined as their elevation position relative to local tidal range, is widely recognized as a key constraint on the horizontal expansion of tidal marsh vegetation^33,34^. To quantify how relative elevation constrains vegetation establishment in restored tidal wetlands, we related restoration-scale tidal marsh vegetation cover to relative elevation. Vegetation cover was defined at the restoration-site scale as the proportion of vegetated tidal marsh area relative to total tidal wetland area (the area of tidal flat and marsh) within the mapped restoration boundary, and is reported as a percentage (0–100%). Time series were summarized at 3-year time steps, and current cover was taken from the most recent time step available for each site. Relative elevation was defined as the percentage position of a site’s mean surface elevation within the local tidal frame, calculated between mean low water spring (MLWS) and mean high water spring (MHWS) tide levels (Supplementary Fig. 7). Site elevations were extracted from the most recent available digital terrain model (DTM) for each site, and tidal range metrics were obtained from the nearest tide gauge; elevation and tidal datums were harmonized before computing relative elevation^16^. We did not explicitly exclude tidal creek beds when extracting mean site elevation from DTMs. However, for the subset of sites with mapped tidal creek vectors, a technical check showed that excluding creeks made little difference to mean elevation. Removing a 1 m buffer around mapped creeks increased mean elevation by only 0.052 m on average (median 0.035 m; *n* = 17).

We fitted a generalized additive model (GAM) with a beta error distribution and logit link, which is appropriate for continuous proportional vegetation cover bounded between 0 and 1. To handle exact 0 and 1 values, we applied a Smithson–Verkuilen adjustment (a linear transformation that slightly compresses the data into the open interval (0, 1)) prior to model fitting. To capture potential nonlinearity in the elevation–cover relationship, relative elevation was modelled using a penalized cubic regression spline, with smoothing complexity selected by restricted maximum likelihood (REML). To test whether commonly cited contextual factors add explanatory power beyond elevation, we treated year since restoration and restoration area (log-transformed) as candidate covariates and evaluated their support via model comparison. We fitted candidate models, including all combinations of these covariates (with the elevation smooth included in all models), and compared them using AIC. The best-supported model retained only the relative-elevation smooth term; adding year since restoration and/or restoration area did not reduce AIC, and their estimated effects were weak (Supplementary Tables 6 and 9).

We assessed predictive stability using repeated random-split cross-validation (80% training, 20% testing; 10 repeats) and summarized prediction error using RMSE (i.e., the typical difference between predicted and observed marsh cover on the 0–1 scale; median = 0.199), which was 12.6% lower than a mean-only reference model. In addition, we refitted the final GAM using nonparametric bootstrap resampling of sites (B = 500 bootstrap resamples) to evaluate the robustness of the inferred elevation smooth. The elevation effect was highly consistent across resamples, with the smooth term remaining statistically significant in 99.8% of bootstrap refits (*P* < 0.05), and we report the corresponding bootstrap uncertainty band for the fitted elevation–cover relationship in Supplementary Fig. 8.

### Sub-site creek drainage efficiency and early-stage marsh colonization

Tidal creek networks are widely regarded as key features that promote drainage while reducing soil salinity and redox stress, thereby creating favorable conditions for early vegetation establishment in restored wetlands^37–39^. To evaluate the influence of tidal creek network efficiency on early marsh colonization, we selected a subset of restoration sites that met two criteria: (i) the mean relative elevation was below 100%, indicating regular exposure to tidal inundation, and (ii) the vegetated tidal marsh cover remained below 80% during the first three years after restoration, ensuring a measurable period of colonization. Accordingly, 17 sites fulfilled these criteria. At each selected site, tidal channel networks were manually digitized from high-resolution Google Earth imagery captured during the early restoration phase. All imagery was standardized to 1-m spatial resolution. Creek segments narrower than 1 m were excluded to maintain consistency across sites. Creek network efficiency was quantified using two established hydrological metrics that capture drainage connectivity and spatial accessibility: drainage density (D), calculated as the total length of tidal channels normalized by watershed area (m^−1^), and mean unchanneled path length (UPL), representing the average straight-line distance from each marsh platform pixel to the nearest tidal creek or the seaward marsh boundary. These indices were calculated at 1 × 1 m resolution for each site, using the restored area boundary as the watershed extent. Drainage density reflects the degree of hydrological connectivity, while UPL represents the efficiency of water movement across the platform. Drainage density and UPL are commonly used indices to quantify the efficiency of tidal creek networks in regulating water exchange across marsh platforms^66,67^. A higher drainage density and lower UPL indicate a more effective tidal channel network.

To test the association between tidal channel structure and vegetation establishment, we applied linear regression models to examine the relationship between each creek efficiency metric (D and UPL) and the early colonization rate of vegetated tidal marsh (% yr^−1^) during the first three post-restoration time intervals (9 years in total). Given the relatively small sample size in the sub-site creek-efficiency analysis, we also performed a leave-one-out sensitivity analysis to assess whether the fitted relationships were disproportionately influenced by any single observation. We sequentially excluded one site at a time and refitted the models to evaluate their stability. Across all iterations, the relationship with drainage density remained positive and significant (slope: 0.027–0.054; *R*²: 0.39–0.65; *P* < 0.01), indicating that the observed trend was robust to the removal of any single site.

### Joint hierarchical modelling of cross-scale restoration processes

To integrate hydro-geomorphological drivers operating at different spatial scales and to evaluate whether distinct recovery processes are statistically coupled across restoration sites, we implemented a joint hierarchical modelling framework. This approach allowed us to synthesize evidence from analyses conducted at the regional, site, and sub-site scales within a single model structure, while explicitly accounting for shared site-level variation and uneven data availability among response variables^68^.

Building on this joint framework, we compiled a dataset that combined restoration performance metrics with hydro-geomorphological drivers operating at regional, site, and sub-site scales across managed realignment projects. Three complementary response variables were defined to represent different dimensions of restoration: (i) tidal wetland area non-loss; (ii) current vegetation cover; and (iii) the early-stage vegetation colonization rate. Although these metrics were analyzed separately in the preceding scale-specific analyses, they capture different but potentially interrelated aspects of restoration trajectories and were therefore integrated within a single hierarchical framework^69^.

Key hydro-geomorphological drivers were selected to represent dominant physical controls at each spatial scale, including regional suspended sediment availability (TSM), site-scale relative elevation within the tidal frame, and sub-site tidal creek density. Prior to modelling, all continuous predictors were mean-centered and scaled to unit variance (z-score standardization) to facilitate comparison of effect sizes and improve model convergence and numerical stability. Because not all predictors were available for all restoration sites, missing predictor values were handled using a missing-indicator approach. Specifically, missing values of standardized predictors were replaced with zero (corresponding to the mean of the standardized distribution), and a binary indicator variable (missing = 1, observed = 0) was included for each predictor to explicitly account for potential bias associated with incomplete information. This approach allowed us to retain all sites in the joint hierarchical analysis, rather than excluding sites with any missing predictor values.

Using the standardized, gap-tolerant dataset, we fitted a multivariate Bayesian hierarchical model in MCMCglmm^68^. Each response was modelled using an appropriate likelihood (binary for tidal wetland non-loss, Gaussian for logit-transformed vegetation cover and early-stage colonization rate). We specified an unstructured site-level covariance matrix to estimate correlations among tidal wetland non-loss, post-restoration vegetation cover, and early-stage colonization rate, thereby testing whether these restoration dimensions co-vary across sites. To ensure that cross-response dependence was attributed to shared site-level influences rather than to residual-level coupling, between-trait residual covariances were fixed to zero.

Models were fitted in MCMCglmm using Markov chain Monte Carlo sampling (200,000 iterations; first 50,000 discarded; retaining every 100th iteration). We verified that parameter estimates were stable across the sampling run and that the retained samples were sufficient for inference. Results are reported as posterior means with 95% credible intervals (Supplementary Table 2 and Supplementary Fig. 9).

To evaluate the robustness of our primary joint model, we performed three complementary sensitivity analyses focusing on missing data handling, prior specifications, and model structure. Specifically, we first restricted the multivariate joint analysis to a complete-case dataset, then re-fitted the model utilizing an alternative prior for the site-level random-effect covariance structure, and developed an extended multivariate model incorporating a secondary hydro-geomorphological covariate for each response variable to account for potential cross-scale constraints. Across these analyses, posterior estimates of the primary effects remained broadly consistent with the main model, though uncertainty surrounding wetland persistence increased marginally in certain scenarios. Comprehensive details are available in Supplementary Note 1 and Supplementary Table 4.

By jointly modelling multiple recovery dimensions within a hierarchical framework, this approach moves beyond scale-separated analyses and allows us to evaluate how different restoration processes relate to one another across sites. It provides a unified, scale-explicit way to assess how hydro-geomorphological constraints shape tidal wetland restoration trajectories under managed realignment.

### Mapping global tidal wetland restoration potential

Having identified the key hydro-geomorphological controls on tidal wetland restoration outcomes across multiple spatial scales, we developed a global, threshold-based assessment framework to estimate the restoration potential of tidal wetlands lost to direct anthropogenic activities and to explicitly quantify associated uncertainty.

To this end, we integrated multiple global geospatial datasets and applied a stepwise filtering approach. We first identified tidal wetlands lost primarily due to direct human activities, ensuring that candidate areas remain physically above water and potentially restorable through managed realignment. Building on model-informed thresholds for sediment availability and relative elevation, we then screened these candidate areas to distinguish zones likely to maintain tidal wetlands from those additionally suitable for marsh vegetation establishment. Finally, we evaluated how uncertainty in these hydro-geomorphological thresholds propagates into estimates of global restoration potential using both discrete scenario-based sensitivity analyses and Monte Carlo uncertainty propagation, enabling a spatially explicit assessment of restoration robustness at the global scale.

### Identifying tidal wetlands lost to direct anthropogenic activities

To estimate the global restoration potential of tidal wetlands lost due to anthropogenic activities, we integrated multiple geospatial datasets and applied a stepwise filtering framework. First, we identified areas of tidal wetland loss from 1999 to 2019 using the global dataset^3^, which maps loss due to direct human actions such as conversion to agriculture and aquaculture, urban expansion, and hydrological alterations (e.g., diking and channel diversions), and those driven by sea-level rise and natural coastal processes. To isolate wetlands lost primarily to direct anthropogenic drivers, we intersected this dataset with the DeltaDTM elevation dataset^70^, which represents the elevation of current intertidal zones while excluding permanent open water. Since wetlands lost to indirect drivers such as coastal processes and climate change have typically transitioned into permanent open water, they are no longer represented in the DeltaDTM dataset. As a result, our spatial overlap approach effectively excludes these areas and conservatively captures those wetlands historically lost due to direct anthropogenic activities that remain physically above water and are potentially restorable through managed realignment (MR).

### Model-informed threshold definition for restoration screening

Having identified tidal wetlands lost to direct anthropogenic activities that meet the basic criteria for potential managed realignment, we next defined hydro-geomorphological screening criteria to assess where different restoration outcomes are likely to occur under current conditions. Using the fitted loss–no change–gain ordinal model, we predicted restoration outcomes across the observed range of total suspended matter (TSM), with tidal range fixed at its median value to provide a representative hydrodynamic context. Based on these predictions, we identified a sediment availability threshold as the TSM level at which the probability of wetland gain reached 0.5. This gain-50 threshold occurred at approximately 20.37 g m^−3^and was adopted as the primary sediment screening threshold in subsequent global analyses. The close agreement between this model-derived threshold and previously reported sediment requirements for tidal wetland persistence (~20 g m^−3^, Supplementary Table 10)^9,57^ provides independent support for its ecological relevance. Uncertainty in this threshold was quantified using bootstrap resampling, yielding a 95% uncertainty interval of 14.53–26.84 g m^−3^, which was propagated into the global restoration potential assessment.

In parallel, we used the fitted elevation–cover relationship to derive an operational relative-elevation threshold for screening where tidal marsh establishment is likely following MR. We defined a functional establishment criterion as the relative elevation at which predicted vegetation cover reaches a high-cover state (cover ≥ 80%). The primary elevation threshold was identified as the lowest relative elevation at which the fitted mean prediction exceeded this criterion, corresponding to 88% of the local tidal range. This threshold lies within the upper tidal frame, where reduced inundation frequency favors persistent marsh vegetation, consistent with empirical, species-level, and process-based evidence linking marsh establishment to higher relative elevations within the tidal frame (Supplementary Table 11)^41,71–73^. To characterize uncertainty around this estimate, we additionally identified a conservative threshold based on the lower uncertainty bound (101%), representing elevations at which high vegetation cover is expected across the full range of model uncertainty, and an optimistic threshold based on the upper uncertainty bound (70%), representing the earliest elevation at which high cover becomes plausible (Supplementary Fig. 10). This elevation envelope (70–101% of the local tidal frame) was subsequently used in sensitivity analyses to assess how uncertainty in elevation thresholds propagates into estimates of restoration potential.

### Sensitivity analysis of threshold-based restoration potential

Following the identification of hydro-geomorphological thresholds and their associated uncertainty ranges, we quantified global tidal wetland restoration potential using a stepwise screening framework and evaluated the sensitivity of restoration outcomes to uncertainty in threshold selection.

We first applied the model-derived sediment availability threshold (TSM = 20.4 g m^−3^) to a global, multi-decadal total suspended matter (TSM) dataset from the GlobColour archive. This approach allowed us to identify areas where tidal wetlands are likely to be maintained following managed realignment under current conditions. Within these potentially maintainable wetland areas, we further distinguished zones likely to recover as vegetated tidal marshes from those more likely to persist as unvegetated tidal flats using the relative elevation threshold. Global relative elevation was calculated by normalizing DeltaDTM elevations using locally derived tidal range estimates constructed from the TPXO tidal model (https://www.tpxo.net), such that elevation was expressed as a fraction of the local tidal frame. Based on the fitted elevation–vegetation cover relationship, areas with relative elevations exceeding 88% of the local tidal range were classified as having high potential for marsh vegetation establishment, while areas below this threshold were considered more likely to support persistent tidal flats.

To evaluate how uncertainty in sediment and elevation thresholds propagates into estimates of restoration potential, we conducted a two-level sensitivity analysis. At the first level, we implemented a discrete, scenario-based sensitivity analysis using alternative but ecologically plausible threshold values derived from model uncertainty. For sediment availability, we used the lower, central, and upper threshold values of 14.53, 20.37, and 26.84 g m^−3^, corresponding to the 95% uncertainty interval around the primary TSM threshold. For relative elevation, we used three thresholds representing optimistic, central, and conservative conditions (70%, 88%, and 101% of the local tidal frame). These sediment and elevation thresholds were combined factorially to generate nine discrete screening scenarios (3 × 3). For each scenario, we quantified (i) the total area where tidal wetlands are expected to be maintained and (ii) the subset of this area where marsh vegetation establishment is likely. Comparing results across these nine scenarios provided an intuitive range of possible restoration outcomes and allowed us to bracket the minimum, intermediate, and maximum estimates of global wetland maintenance and vegetation establishment potential (Supplementary Table 5).

At the second level, we conducted a Monte Carlo–based uncertainty propagation analysis to characterize restoration uncertainty across the continuous threshold space. Sediment thresholds were randomly sampled from a uniform distribution spanning 14.53–26.84 g m^−3^, and elevation thresholds were sampled from a uniform distribution spanning 70–101% of the local tidal frame. For each Monte Carlo iteration (n = 100), the threshold-based screening procedure was repeated at the grid-cell level. This approach generated distributions of restoration outcomes for each spatial unit, from which we derived multiple complementary uncertainty metrics. These included the standard deviation (SD, km²), representing the absolute magnitude of variability across simulations; the coefficient of variation (CV = SD/mean), representing relative uncertainty normalized by the mean outcome; and the 95% uncertainty interval width (CI width, km²), quantifying the absolute range between the most optimistic and most conservative outcomes (see Supplementary Fig. 3). Together, these metrics provide spatially explicit measures of the robustness and sensitivity of restoration predictions, identifying regions where outcomes are stable across plausible threshold values versus regions where predictions are highly sensitive to threshold uncertainty.

Following this framework, we mapped global potential tidal wetland restoration areas, including both tidal flats and tidal marshes, at a 2-degree resolution, and produced spatially explicit maps of uncertainty associated with threshold-based screening. We further quantified the proportion of restorable wetlands relative to historically lost tidal wetlands for each continent. These results were evaluated against the 30% ecosystem restoration target proposed by the Kunming–Montreal Global Biodiversity Framework to identify priority regions where MR could make meaningful contributions toward global restoration goals. All analyses were performed in R version 4.4.0^74^.

## Supplementary information

Supplementary Information

Transparent Peer Review file

## Data Availability

The primary datasets generated in this study have been deposited in the Figshare database under [10.6084/m9.figshare.31143697]^75^. The tidal wetland restoration site data used in this study are available in the OMReg, NOAA Restoration Atlas [https://www.fisheries.noaa.gov/resource/map/restoration-atlas], and ACRN databases. The Landsat imagery used in this study is available in the U.S. Geological Survey database [http://earthexplorer.usgs.gov] and the Google Earth Engine database [https://earthengine.google.com]. The high-resolution historical imagery used in this study is available in Google Earth. The surface total suspended matter (TSM) data used in this study are available in the GlobColour archive database [http://hermes.acri.fr/]. The elevation data for European sites used in this study are available in the Flanders mapping portal for Belgium [https://www.vlaanderen.be/], the IGN portal for France [https://geoservices.ign.fr/], the BKG metadata portal for Germany [https://mis.bkg.bund.de/], the CNIG download portal for Spain [https://centrodedescargas.cnig.es/], the UK Environment Agency database via [https://data.gov.uk] for the UK, and the Google Earth Engine database under the catalog AHN Netherlands 0.5 m DEM for the Netherlands [https://developers.google.com/earth-engine/datasets/catalog/AHN_AHN2_05M_INT]. The elevation data for sites in the Americas used in this study are available in the Google Earth Engine database under the catalogs Canadian Digital Elevation Model for Canada [https://developers.google.com/earth-engine/datasets/catalog/NRCan_CDEM] and USGS 3DEP 1 m National Map for the United States [https://developers.google.com/earth-engine/datasets/catalog/USGS_3DEP_1m]. The elevation data for Australian sites used in this study are available in the Google Earth Engine database under the catalog Australian 5 M DEM https://developers.google.com/earth-engine/datasets/catalog/AU_GA_AUSTRALIA_5M_DEM. The local tidal data used in this study are available in the Reeds Nautical Almanac 2020^76^, and in the NOAA Tides & Currents [https://tidesandcurrents.noaa.gov/], Fisheries and Oceans Canada [https://tides.gc.ca/en], and Australian Hydrographic Office [https://www.hydro.gov.au/prodserv/publications/ahp11.htm] databases. The global tidal wetland loss data used in this study are available in the Global Tidal Wetland Change Dataset database [https://developers.google.com/earth-engine/datasets/catalog/JCU_Murray_GIC_global_tidal_wetland_change_2019]. The TPXO tidal model data used to derive the global tidal range in this study are available at [https://www.tpxo.net/home]. The global wetland elevation data used in this study are available in the DeltaDTM v1.1 database under accession code 10.4121/21997565.v4 [10.4121/21997565.v4]. The coastline base maps used in the figures are available from Natural Earth [https://www.naturalearthdata.com/].

## Code availability

The custom code used to analyze the data in this study has been deposited in the Figshare database [10.6084/m9.figshare.31143697].
