# Supplementary material for: Hydro-geomorphological drivers across scales shape the trajectory of coastal wetland restoration
Source: Nat Commun. 2026 Apr 22;17:5594. doi: 10.1038/s41467-026-71992-x (PMC13315590; doi:10.1038/s41467-026-71992-x)
Supplement: Supplementary file 1 — Supplementary Information [file 41467_2026_71992_MOESM1_ESM.pdf]

Hydro-geomorphological drivers across scales shape the trajectory of coastal wetland restoration

Supplementary Information

Junlin Ren, Sikai Wang, Tingting Zhang, Jun Ma, Ting Zhang, Chuyu Cheng, Kang Zhang, Changlin Xu, Johan van de Koppel, Daphne van der Wal, Tjeerd J. Bouma, Fangyan Cheng, Ping Zhuang, Feng Zhao*

* Corresponding author. Email: zhaof@ecsf.ac.cn

Supplementary Note 1 Bayesian implementation, MCMC settings, and convergence assessment

The multivariate joint hierarchical Bayesian models were fitted in MCMCglmm in R. Each model was run for 200,000 iterations, with a burn-in period of 50,000 iterations and a thinning interval of 100, yielding 1,500 retained posterior samples for inference. For the main model, we used weakly informative priors for the residual and site-level random-effect covariance structures.

Convergence and sampling efficiency were assessed using both graphical and numerical diagnostics. Visual inspection of the trace plots for the focal fixed effects indicated adequate mixing and no obvious long-term drift (Supplementary Fig. 9). Effective sample sizes (ESS) for the focal parameters in the main model ranged from 1062 to 1500, and lag-1 autocorrelation ranged from 0.015 to 0.170, indicating that posterior sampling was sufficient for robust inference (Supplementary Table 2).

Sensitivity Analyses

To evaluate the robustness of our inference to assumptions about missing-data treatment, prior specification, and model structure, we conducted three complementary sensitivity analyses (Supplementary Table 4).

1. Complete-case analysis

To assess whether inference depended on the missing-indicator approach used in the focal model, we repeated the multivariate joint analysis using a strict complete-case dataset, retaining only observations with complete records for the three focal covariates (TSM, relative elevation, and creek density). In this sensitivity analysis, the missing-indicator terms were removed from the model, while the prior specification and MCMC settings were otherwise kept the same as in the main analysis. Although the reduced sample size increased uncertainty relative to the main model, the focal effects remained directionally consistent with the primary analysis. In the complete-case model, the posterior mean effect of TSM on wetland persistence was 125.26 (95% CI = [24.70, 231.04], pMCMC = 0.007), the effect of relative elevation on marsh cover was 1.41 (95% CI = [0.28, 2.60], pMCMC = 0.013), and the effect of

creek density on early-stage colonization rate was 3.89 (95% CI = [2.09, 5.72], pMCMC < 0.001).

2. Prior sensitivity

To assess whether posterior inference depended materially on prior choice, we re-fitted the model under an alternative prior specification for the site-level random-effect covariance structure. Specifically, we increased the relevant prior hyperparameter from 4 to 10, while leaving the residual prior unchanged. Under this alternative prior specification, the posterior mean effect of TSM on wetland persistence was 77.41 (95% CI = [-1.92, 158.38], pMCMC = 0.055), the effect of relative elevation on marsh cover was 1.95 (95% CI = [1.11, 2.77], pMCMC < 0.001), and the effect of creek density on early-stage colonization rate was 2.95 (95% CI = [1.36, 4.51], pMCMC = 0.004). Posterior summaries for the focal effects were therefore qualitatively unchanged under this alternative prior specification, indicating that the substantive conclusions were not sensitive to this prior choice.

3. Extended model with secondary covariates

To assess the structural robustness of the primary joint model, we fitted an extended multivariate model that added a secondary hydro-geomorphological covariate to each response, reflecting potential cross-scale constraints. In addition to the primary predictors (TSM for wetland persistence, relative elevation for marsh cover, and creek density for colonization rate), we included: (i) relative elevation as an additional covariate for the wetland persistence response, (ii) regional sediment supply (TSM) as an additional covariate for the marsh cover response, and (iii) relative elevation as an additional covariate for the colonization rate response.

In this extended model, relative elevation remained positively associated with marsh cover (posterior mean = 1.97, 95% CI = [1.09, 2.82], pMCMC < 0.001), and creek density remained positively associated with early-stage colonization rate (posterior mean = 2.71, 95% CI = [0.45, 4.89], pMCMC = 0.024). The posterior mean effect of TSM on wetland persistence also remained positive (posterior mean = 66.07), although the 95% credible interval overlapped zero (95% CI = [-8.73, 147.08], pMCMC = 0.075). The added secondary covariates did not show clear associations

with the corresponding responses. Overall, the extended-model results were broadly consistent with the main conclusions across all three responses, demonstrating that inference on the main drivers was not highly sensitive to the inclusion of these secondary covariates.

Taken together, these diagnostics and sensitivity analyses indicate that the main conclusions were not strongly driven by prior choice, the inclusion of missing-indicator terms, or the addition of secondary covariates. Nevertheless, because the missing-indicator approach is primarily a pragmatic solution, bias under non-random missingness cannot be fully excluded, although the directional consistency across our sensitivity analyses provides confidence in the primary conclusions.

Supplementary Table 1 | Ordinal logistic regression linking suspended sediment availability and tidal range to wetland trajectory class. Wetland trajectory (loss–stable–gain; ordered factor) was modelled as a function of suspended sediment availability (TSM) and tidal range using a proportional-odds ordinal logistic regression. Predictors were z-score standardized, so coefficients and odds ratios represent effects per 1 SD increase in each predictor. Effect sizes are reported as log-odds coefficients (β ± SE), Wald z statistics and two-sided P values, and odds ratios (OR = exp[β]) with Wald 95% confidence intervals (exp[β ± 1.96×SE]).

**Predictors**	**β**	**SE**	**Z value**	**P value**	**OR**	**OR 95% CI**
TSM	1.07	0.38	2.86	**0.0043**	2.92	1.40–6.08
Tidal range	-0.34	0.34	-1.024	0.31	0.71	0.37–1.36

Supplementary Table 2 | Fixed effects from the multivariate Bayesian joint hierarchical model. A multivariate Bayesian joint hierarchical model was fitted in MCMCglmm with three responses linked by correlated site random effects. Fixed-effect estimates are reported on the scale used for each response: a latent logit scale for wetland persistence (y1; categorical), the identity scale of the logit-transformed marsh cover (y2; Gaussian), and the identity scale for early-stage colonization rate (y3; Gaussian). Predictors were z-score standardized (per 1 SD); creek density was additionally rescaled (z/2) prior to modelling. Values are posterior means with 95% credible intervals (CI), effective sample sizes (ESS), and posterior MCMC P values (pMCMC).

Response	Predictor	Posterior mean	95% CI	ESS	pMCMC
**Wetland persistence**	TSM	75.63	[1.55, 161.58]	1220	**0.047**
**Marsh cover**	Relative elevation	1.95	[1.05, 2.76]	1500	**<0.001**
**Early-stage colonization rate**	Creek density	3.03	[1.14, 4.84]	1062	**0.0013**

Supplementary Table 3 | Between-site posterior correlations indicate limited coordination among restoration dimensions. Across sites, we quantified how restoration dimensions co-varied by estimating correlations among the site random effects for wetland persistence, marsh cover, and early-stage colonization rate using an unstructured covariance matrix. Values are posterior mean correlations (r) derived from the posterior mean covariance matrix. Positive values indicate that sites performing better on one dimension also tend to perform better on the other, whereas negative values suggest trade-offs across dimensions.

**Response pair**	**Posterior mean correlation (r)**
Wetland persistence & Marsh cover	0.16
Wetland persistence & Early-stage colonization rate	−0.10
Marsh cover & Early-stage colonization rate	−0.27

Supplementary Table 4 | Results of sensitivity analyses for the multivariate Bayesian joint hierarchical model. Values are posterior means with 95% credible intervals (CI), effective sample sizes (ESS), and posterior MCMC P values (pMCMC) for the focal fixed effects across three alternative model specifications. In the extended model, secondary covariates (relative elevation for wetland persistence, TSM for marsh cover, and relative elevation for early-stage colonization rate) were included but are not displayed here, as all their main effects were non-significant (all $P > 0.48$).

Sensitivity Model	Response	Predictor	Posterior mean	95% CI	ESS	pMCMC
**Complete-case analysis**	Wetland persistence	TSM	125.26	[24.70, 231.04]	654	**0.007**
	Marsh cover	Relative elevation	1.41	[0.28, 2.60]	1500	**0.013**
	Early-stage colonization rate	Creek density	3.89	[2.09, 5.72]	1700	**< 0.001**
**Prior sensitivity**	Wetland persistence	TSM	77.41	[-1.92, 158.38]	1500	**0.055**
	Marsh cover	Relative elevation	1.95	[1.11, 2.77]	1500	**< 0.001**
	Early-stage colonization rate	Creek density	2.95	[1.36, 4.51]	856	**0.004**
**Extended model**	Wetland persistence	TSM	66.07	[-8.73, 147.08]	1259	**0.075**
	Marsh cover	Relative elevation	1.97	[1.09, 2.82]	1500	**< 0.001**
	Early-stage colonization rate	Creek density	2.71	[0.45, 4.89]	613	**0.024**

Supplementary Table 5 | Potentially restorable tidal wetland and marsh areas (km^2) under different thresholds of total suspended matter (TSM) and relative elevation.

TSM threshold ($g\ m^{-3}$)	Elevation threshold (% of tidal range)	Potentially restorable tidal wetland (km^2)	Potential marsh area (km^2)	Potential tidal flat area (km^2)
14.53	70	1308.52	638.64	669.88
14.53	88	1308.52	550.57	757.96
14.53	101	1308.52	466.50	842.02
20.37	70	923.99	450.41	473.57
20.37	88	923.99	388.57	535.42
20.37	101	923.99	325.52	598.47
26.84	70	567.83	290.48	277.35
26.84	88	567.83	252.11	315.72
26.84	101	567.83	210.49	357.34

Supplementary Table 6 | Summary of GAM relating relative elevation, restoration age, and site area to restoration-scale marsh cover.

Model	Term	Estimate	SE	EDF	*P*-value
Marsh cover (beta GAM)	Intercept	33.68	35.33		0.42
	Restoration age	-0.016	0.018		0.37
	Restoration area	-0.074	0.13		0.55
	**Relative elevation**			1.30	**0.00050**
Model fit	adj. $R^2 = 0.20$				
Sample size	56				

Supplementary Table 7 | Metadata of managed realignment (MR) restoration sites across Europe, North America, and Oceania.

Restoration site	Target area (ha)	Latitude	Longitude	Year started	Country
Abbotts_Hall	84	51.78	0.85	2002	UK
Aber_de_Crozon	90	48.24	-4.43	1980	France
Alkborough	370	53.69	-0.68	2006	UK
Allfleets_Marsh	133	51.62	0.84	2006	UK
Bildtpollen	60	53.33	5.71	2009	Netherlands
Billwerder_Insel	20	53.51	10.07	2008	Germany
Bleadon_Levels	13	51.31	-2.99	2001	UK
Chowder_Ness	15	53.69	-0.48	2006	UK
Devereaux_Farm	15	51.86	1.25	2010	UK
Fingringhoe_Wick	22	51.84	0.98	2015	UK
Freiston	66	52.96	0.09	2002	UK
Greatham	40	54.63	-1.22	2013	UK
Hahnöfer_Sand	104	53.55	9.71	2002	Germany
Hauener_Hooge	80	53.52	7.08	1994	Germany
Hesketh_Out_Marsh_East	160	53.73	-2.87	2017	UK
Hesketh_Out_Marsh_West	180	53.72	-2.89	2008	UK
Holwerder_Zomerpolder	28	53.38	5.90	1989	Netherlands
Jubilee_Marsh	165	51.60	0.86	2015	UK
Karrendorfer_Wiesen	350	54.16	13.39	1993	Germany
Ketenisseschor	36	51.28	4.31	2002	Belgium
Kleinensieler_Plate	58	53.46	8.48	2000	Germany
Kreetsand	26	53.50	10.05	1999	Germany
Langeooger_Sommerpolder	215	53.75	7.54	2004	Germany
Lantern_Marsh	29	52.13	1.59	1999	UK
Lütetsburger_Sommerpolder	15	53.67	7.25	1982	Germany
Meddat_Marsh	25	57.74	-4.03	2003	UK
Medmerry	302	50.75	-0.82	2013	UK
Noard_Fryslân_Bûtendyks	135	53.34	5.75	2001	Netherlands
Orplands	38	51.72	0.87	1995	UK
Paardeschor	12	51.33	4.25	2004	Belgium
Paull_Holme_Strays	80	53.71	-0.22	2003	UK
Pepelower	120	54.05	11.59	2002	Germany
Pillmouth	13	51.00	-4.18	2001	UK
Polder_Friedrichshagen	90	54.08	13.51	1999	Germany
Polder_Roggow	40	54.08	11.63	2002	Germany
Salt_Fleet_Flats_Reserve	65	51.48	0.54	2016	UK
Skinflats	11	56.06	-3.73	2018	UK
Sommerpolder_Wurster_Küste	145	53.76	8.53	2007	Germany
Sophiapolder	77	51.84	4.67	2012	Netherlands
Stanford_Wharf	27	51.50	0.45	2010	UK
Steart_Marsh	262	51.20	-3.03	2014	UK
Tegeler_Plate_Polder	150	53.46	8.51	1997	Germany
Tiengemeten	450	51.74	4.29	2007	Netherlands
Titchwell_Marsh	11	52.97	0.61	2011	UK
Tollesbury	21	51.77	0.84	1995	UK
Trimley_Marsh	17	51.98	1.28	2000	UK
Vega_de_Jaitzubia	23	43.35	-1.81	2004	Spain
Welwick	54	53.65	0.01	2006	UK
Yzer_Mouth	50	51.15	2.74	2001	Belgium
Dry_creek	32	-34.73	138.52	2017	Australia
Mungalla_Station_Wetland	49	-18.71	146.28	2012	Australia
Bay_Of_Fundy	20	45.86	-64.30	2010	Canada
Converse_Marsh	10	45.84	-64.27	2018	Canada
American_Canyon_Salt_Pond_Restoration	478	38.19	-122.30	2009	USA
Blue_Heron_Slough_Mitigation_Bank	132	48.03	-122.17	2018	USA
Crescent_Harbor_Tidal_Reconnection	83	48.30	-122.61	2007	USA
Giacomini_Coastal_Wetlands_Restoration	89	38.07	-122.82	2005	USA
Leque_Island_Estuary_Restoration	97	48.23	-122.39	2019	USA
Lincoln_Park_Wetland_Restoration	10	40.73	-74.09	2009	USA
Magnolia_Marsh_Restoration	38	33.64	-117.97	2009	USA
Mid_Spencer_Island_Dike_Breach	28	48.02	-122.15	2019	USA
Musquash_Marsh_Salt_Marsh	137	45.19	-66.33	2004	USA
Nalley_Island_Dike_Removal_Project	91	47.33	-123.13	2010	USA
Nisqually_Estuary	313	47.08	-122.72	2008	USA
Northeast_Florida_Wetland_Restoration	71	28.57	-80.68	2009	USA
Qwuloolt_Estuary_Restoration	154	48.04	-122.16	2007	USA
Riverside_Ranch_Salt_River_Restoration	133	40.61	-124.30	2013	USA
Skokomish	56	47.34	-123.13	2005	USA
Tillamook_Bay_Southern_Flow_Corridor_Restoration	36	45.47	-123.88	2015	USA

Supplementary Table 8 | Summary of accuracy assessments for classified Landsat images upon which the classification algorithms were built. All validation samples were derived from high-resolution Google Earth imagery captured during the vegetation growing season, spanning from the initial year of restoration through up to 30 years after restoration began.

Restoration site	Restoration age	Year started	Overall accuracy (%)	Kappa coefficient
Skinflats	1	2018	96.00	0.78
Hesketh_Out_Marsh_East	4	2017	96.00	0.88
Nisqually_Estuary	5	2008	98.00	0.79
Medmerry	6	2013	88.00	0.77
Hesketh_Out_Marsh_West	9	2008	94.00	0.70
Orplands	11	1999	94.00	0.76
Welwick	11	2006	94.00	0.88
Yzer_Mouth	15	2001	92.00	0.86
Polder_Friedrichshagen	19	1999	97.00	0.79
Aber_de_Crozon	26	1980	88.00	0.76

Supplementary Table 9 | Candidate GAMs relating relative elevation to restoration-scale vegetation cover. Models are ordered according to their AIC, and ΔAIC denotes the difference between each model and the best-fitting model with the lowest AIC.

Predictors included	AIC	ΔAIC	Weight	logLik
**Relative elevation**	**-102.52**	**0.00**	**0.43**	**54.96**
Relative elevation + restoration age	-101.37	1.14	0.24	55.37
Relative elevation + restoration area	-101.12	1.39	0.22	55.26
Relative elevation + restoration age + restoration area	-99.75	2.76	0.11	55.56

Supplementary Table 10 | Model-derived screening thresholds and literature benchmarks for sediment availability.

Threshold	Source
**20.37 g m^{-3}** (14.53–26.84 g m^{-3})	**This study**
20 mg L^{-1} (= 20 g m^{-3})	Ref.[1]
20 g m^{-3}	Ref.[2]

Supplementary Table 11 | Comparison of elevation thresholds and indicative zones for tidal marsh establishment across studies. This Supplementary Table summarizes operational or indicative elevation thresholds for tidal marsh establishment, expressed as a normalized position within a locally defined tidal frame. Because studies normalize elevation using different tidal datums (e.g., MLWL–MHWL, MHWN–MHWS, or MLWS–MHWS), the reported percentages are not strictly comparable across rows. Instead, they are presented together to highlight a consistent qualitative pattern: marsh vegetation typically establishes toward the upper portion of the tidal frame. Abbreviations: MLWL = mean low water level; MLWS = mean low water spring; MHWN = mean high water neap; MHWL = mean high water level; MHWS = mean high water spring; MHHW = mean higher high water. These datums generally increase in elevation in the following order: MLWS < MLWL < MHWN < MHWL < MHWS < MHHW. Values >100% indicate elevations exceeding the upper datum used to define the tidal frame in that specific study. See main text for references.

Threshold	Source
**Primary threshold: 88% of the local tidal frame (MLWS–MHWS). Sensitivity envelope: 70–101%**	**This study**
76-116% of the local tidal frame (MLWL-MHWL)	Ref.[3]
Around 100% of the local tidal frame (MHWN-MHWS)	Ref.[4]
Around 100% of the local tidal frame (MHWN-MHWS)	Ref.[5]
Close to MHHW	Ref.[6]

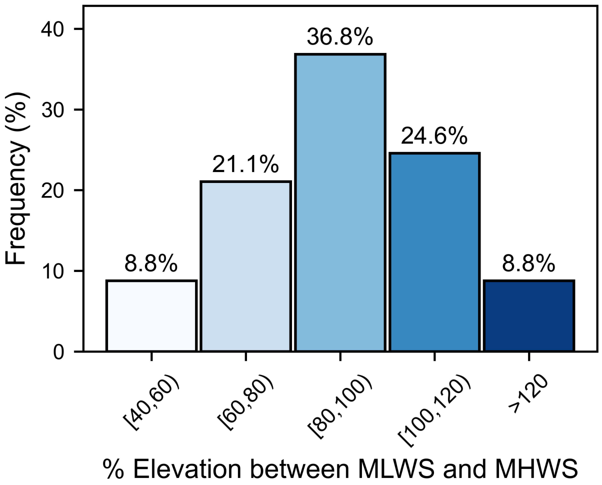

Supplementary Fig. 1 | Frequency distribution of relative elevation across restoration sites. Values above 100% indicate relative elevations exceeding mean high water spring (MHWS) levels, which may still be inundated during extreme tidal events such as highest astronomical tides or storm surges.

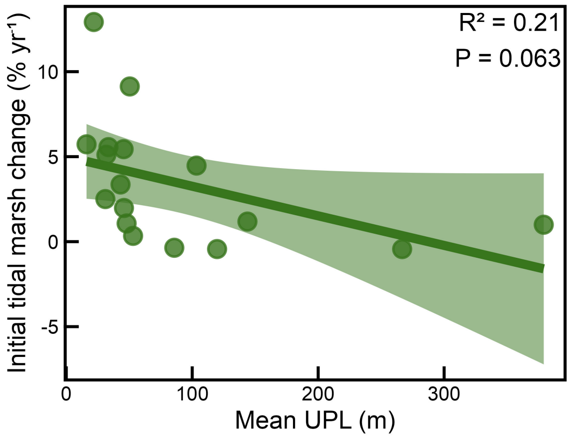

Supplementary Fig. 2 | Relationships between early tidal marsh vegetation colonization rate and mean unchanneled path length (UPL) across restoration sites. Each point corresponds to one restoration site, limited to those with a mean relative elevation below 100% and tidal marsh cover less than 80% within the first three years post-restoration. Shaded areas represent 95% confidence intervals of simple linear regression models.

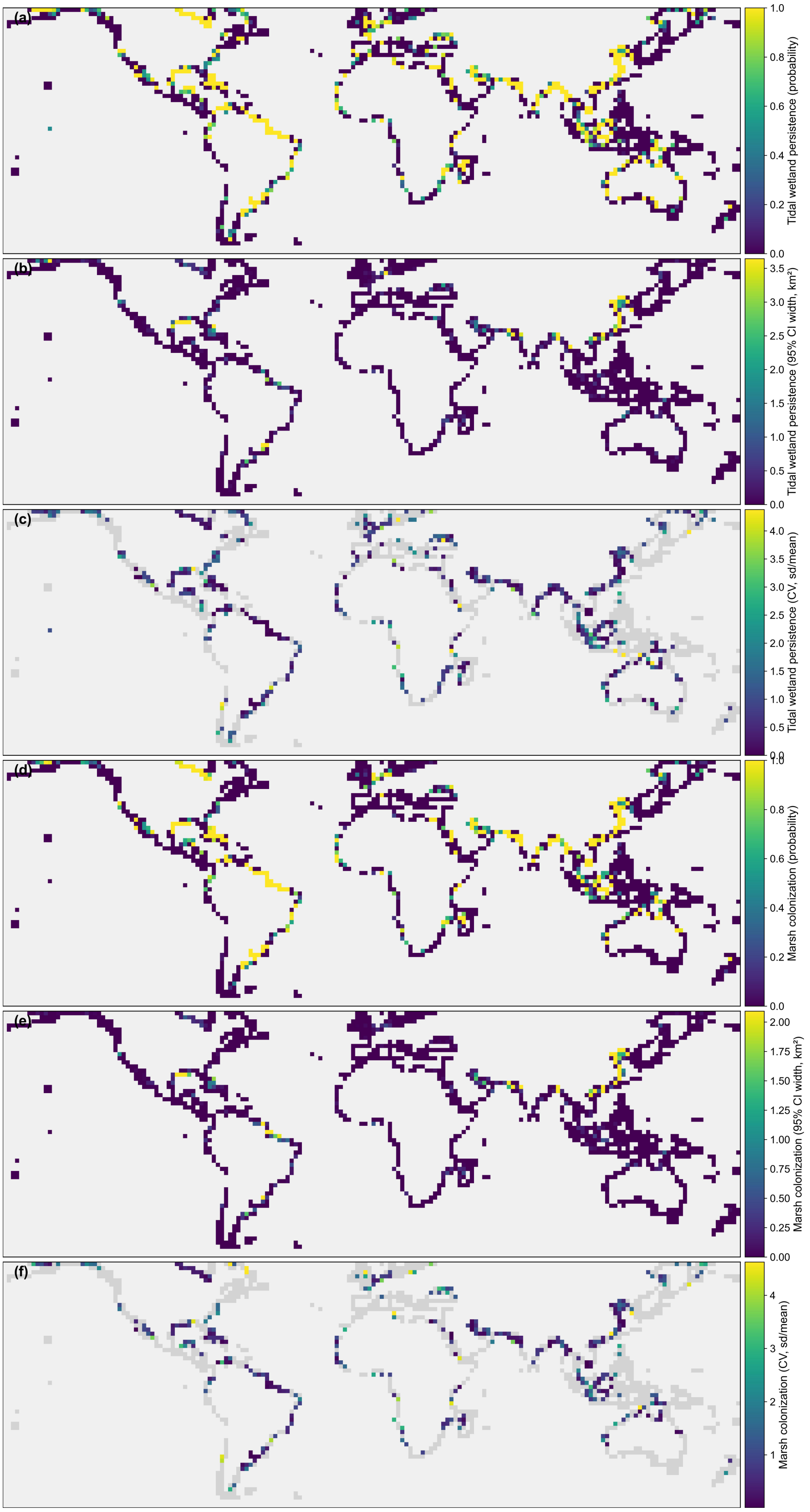

Supplementary Fig. 3 | Spatial propagation of threshold uncertainty in global tidal wetland persistence and marsh colonization. Using Monte Carlo simulations at the grid-cell level (n =100 iterations), we quantified how uncertainty in key sediment (TSM) and relative elevation thresholds propagates into spatial estimates of

(i) tidal wetland persistence and (ii) marsh colonization. Uncertainty was propagated by sampling thresholds from the ranges defined in Supplementary Table 5. Panels show (a) the probability of tidal wetland persistence, (b) the width of the 95% percentile interval (2.5th–97.5th percentiles; km^2) for persistent tidal wetland area, and (c) the coefficient of variation (CV = SD/mean) for persistent area. Panels (d–f) show the corresponding metrics for marsh colonization: (d) colonization probability, (e) the width of the 95% percentile interval for colonized marsh area, and (f) the coefficient of variation for colonized area. Grey cells indicate areas with missing data or undefined CV where the mean area approaches zero.

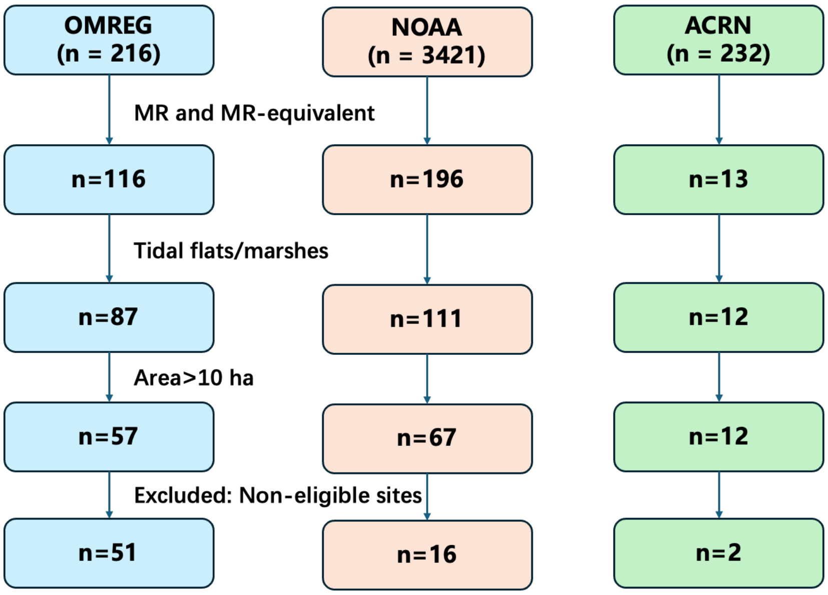

Supplementary Fig. 4 | Workflow for compiling and screening managed realignment (MR) restoration sites from three databases. Candidate coastal restoration projects were collated from OMREG, the NOAA Restoration Atlas, and ACRN. We first retained sites restored through managed realignment (MR) or functionally equivalent tidal-exchange restoration (e.g., deliberate dike breaching or removal). We then restricted the dataset to non-mangrove tidal wetland outcomes (tidal flats and/or tidal marshes) and applied a minimum site-size criterion (area > 10 ha). Finally, non-eligible sites were excluded to obtain the study dataset. Non-eligible sites refer to sites that could not be consistently assessed, including those with indistinct or unmappable boundaries, unclear pre-restoration land use, or insufficient documentation to verify restoration actions and timing.

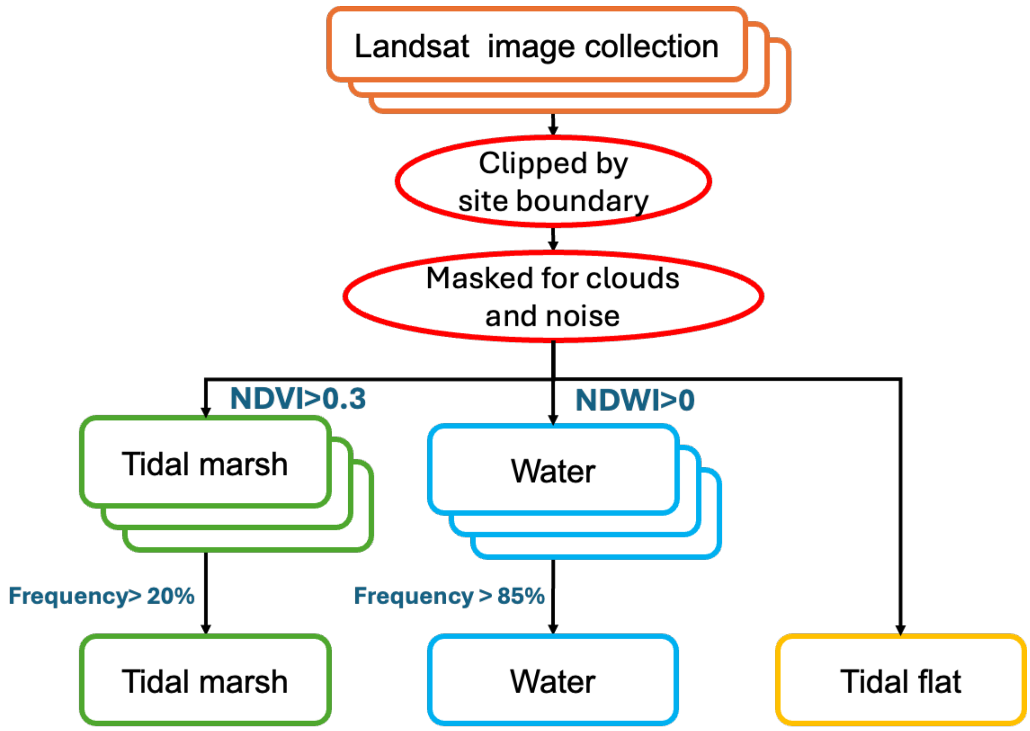

Supplementary Fig. 5 | Flow chart showing the rule-based classification procedure for different wetland types.

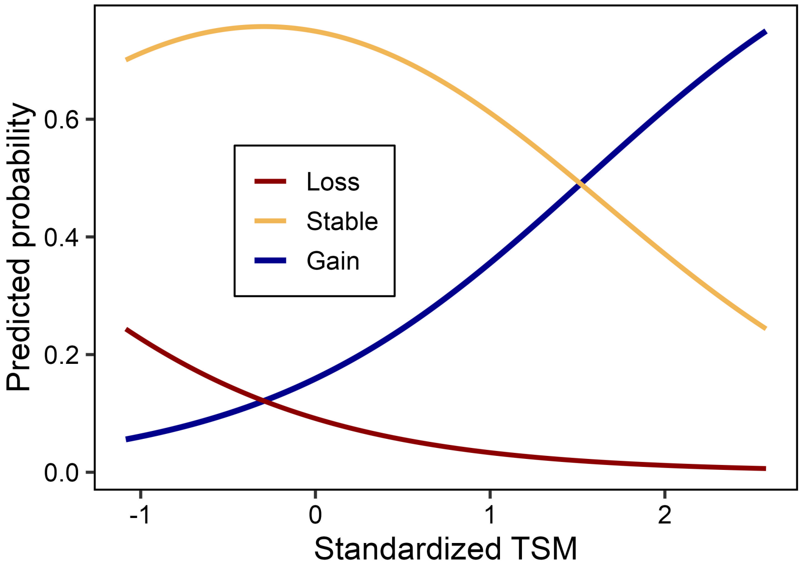

Supplementary Fig. 6 | Predicted wetland trajectory probabilities along the suspended-sediment gradient. Predicted probabilities of three ordered wetland trajectory classes (Loss, Stable, Gain) are shown as a function of standardized total suspended matter (TSM), based on a proportional-odds ordinal logistic regression with tidal range held at its median. Lines indicate the model-predicted class probabilities (red: Loss; yellow: No change; blue: Gain).

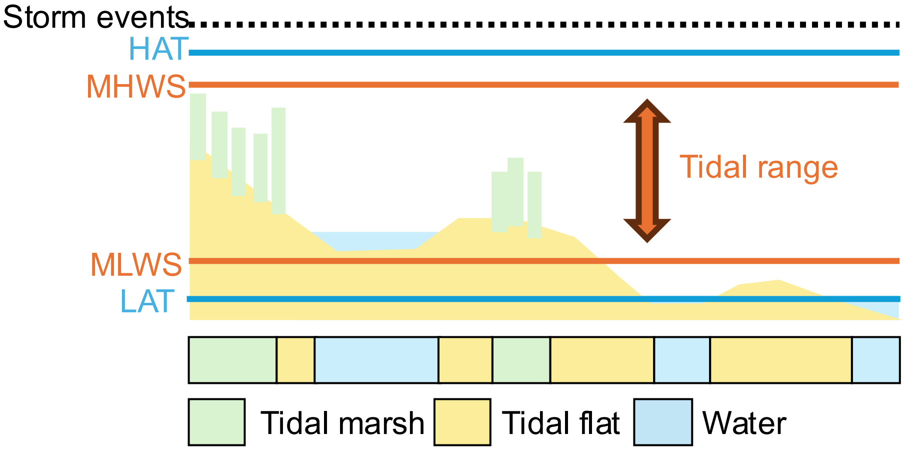

Supplementary Fig. 7 | Schematic illustration of a coastal saltmarsh at different tidal situations. The diagram shows tidal marsh, tidal flat, and open water zones distributed along the elevation gradient. In this study, tidal range is defined as the elevation band between mean high water spring (MHWS) and mean low water spring (MLWS). Areas above MHWS may still be tidally inundated during Highest astronomical tide (HAT) or storm surge events.

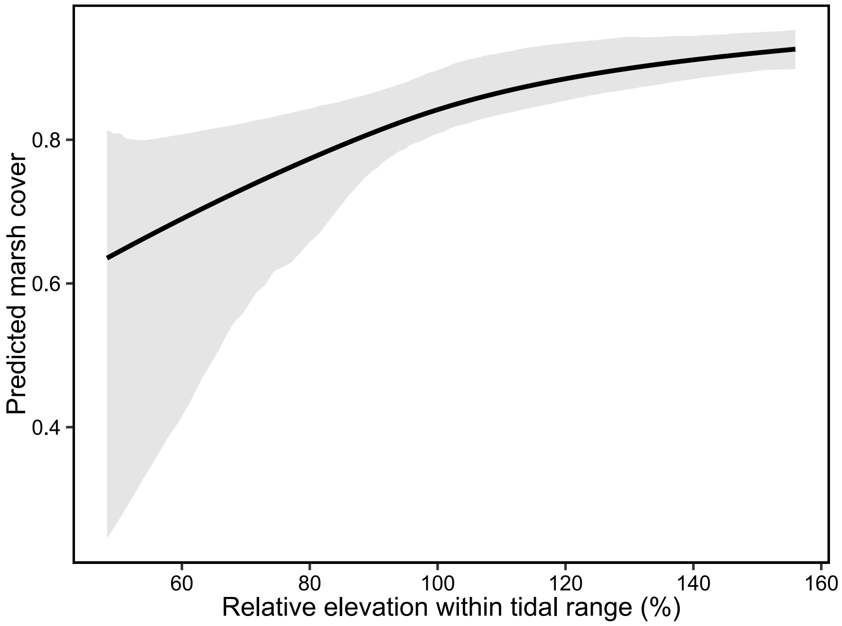

Supplementary Fig. 8 | Relationship between marsh cover and relative elevation. Marsh cover increases with relative elevation (within tidal range). The black line represents the best-fit relationship estimated from the GAM model across all sites. The grey shaded band denotes the 95% bootstrap confidence interval (2.5th–97.5th percentiles) based on 500 resampled refits, representing sampling uncertainty. The effect of elevation was highly robust, reaching statistical significance ($P < 0.05$) in 99.8% of bootstrap iterations.

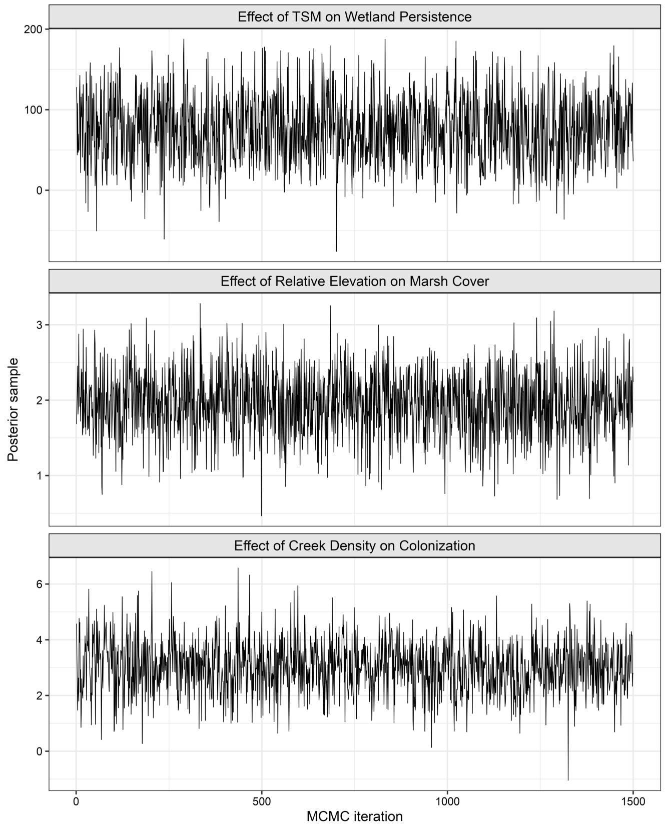

Supplementary Fig. 9 | Trace plots for the three focal fixed effects in the multivariate Bayesian joint hierarchical model. Shown are posterior samples for the effect of total suspended matter (TSM) on wetland persistence, the effect of relative elevation on marsh cover, and the effect of creek density on early-stage colonization rate across retained MCMC iterations. The well-mixed stationary patterns with no long-term drift indicate good convergence and adequate posterior sampling.

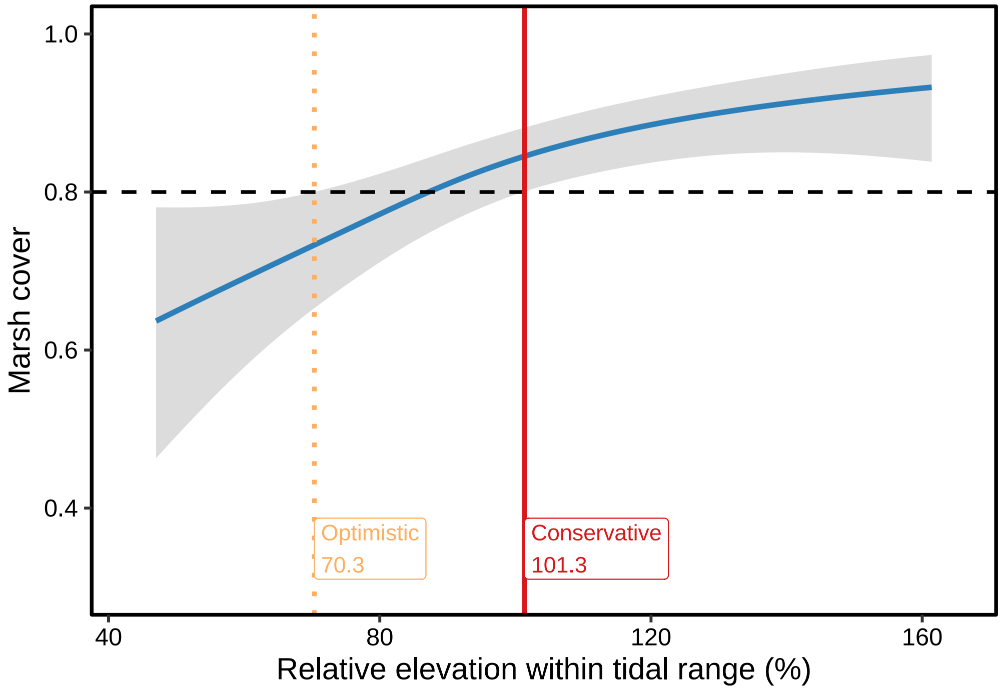

Supplementary Fig. 10 | Elevation thresholds for marsh establishment derived from the elevation–cover relationship. Marsh cover increases with relative elevation within the tidal range. The blue line shows the fitted relationship, and the grey band indicates the 95% uncertainty interval. The horizontal dashed line marks the vegetation-cover criterion used to define successful establishment (cover = 0.8). Vertical lines indicate the inferred elevation thresholds at which predicted marsh cover reaches this criterion: the optimistic threshold (orange, dotted) and the conservative threshold (red, solid), with values annotated on the plot.

Supplementary References

1. Kirwan ML, et al. Limits on the adaptability of coastal marshes to rising sea level. *Geophys. Res. Lett.* **37**, L23401 (2010).
2. Liu Z, Fagherazzi S, Cui B. Success of coastal wetlands restoration is driven by sediment availability. *Commun. Earth Environ.* **2**, 44 (2021).
3. Wang C, et al. Different coastal marsh sites reflect similar topographic conditions under which bare patches and vegetation recovery occur. *Earth Surf. Dyn.* **9**, 71-88 (2021).
4. Mossman HL, Davy AJ, Grant A, Elphick C. Does managed coastal realignment create saltmarshes with 'equivalent biological characteristics' to natural reference sites? *J. Appl. Ecol.* **49**, 1446-1456 (2012).
5. Sullivan MJP, Davy AJ, Grant A, Mossman HL. Is saltmarsh restoration success constrained by matching natural environments or altered succession? A test using niche models. *J. Appl. Ecol.* **55**, 1207-1217 (2018).
6. Stralberg D, et al. Evaluating tidal marsh sustainability in the face of sea-level rise: a hybrid modeling approach applied to San Francisco Bay. *PLoS ONE* **6**, e27388 (2011).
