# Supplementary material for: Hydro-geomorphological drivers across scales shape the trajectory of coastal wetland restoration
Source: Nat Commun. 2026 Apr 22;17:5594. doi: 10.1038/s41467-026-71992-x (PMC13315590; doi:10.1038/s41467-026-71992-x)
Supplement: Supplementary file 2 — Transparent Peer Review file [file 41467_2026_71992_MOESM2_ESM.pdf]

nature portfolio

Peer Review File

Hydro-geomorphological drivers across scales shape the trajectory of coastal wetland restoration

Corresponding Author: Dr Feng Zhao

This file contains all reviewer reports in order by version, followed by all author rebuttals in order by version.

Version 0:

Reviewer comments:

Reviewer #1

(Remarks to the Author)
Dear Authors,
Thank you for the opportunity to review your manuscript titled "Hydro-geomorphological drivers across scales shape the trajectory of coastal wetland restoration." Your work presents a valuable multi-scale framework for assessing restoration success in managed realignment (MR) projects and contributes meaningfully to the global discourse on Nature-based Solutions (NbS).
After careful consideration, I offer the following comments and suggestions to strengthen the manuscript:
Major Comments
1. Geographic and Ecosystem Scope
While the manuscript provides a robust analysis of 49 MR sites in Europe, the exclusive reliance on the OMREG database limits the geographic scope. The Restoration efforts in North America, Australia, and Africa are notably absent, despite their relevance to global restoration targets. The statement that "over 90% of documented MR projects globally are located in Europe" is not quite right as there are many cases available in Northern America (e.g., NOAA) and Australia (Geoscience Australia).
Additionally, mangrove systems—critical coastal wetlands in tropical regions—are not considered, which restricts the ecological applicability of the framework.
Recommendation: A comprehensive literature search would improve the credibility of the research. In particular, any study on coastal wetland much include mangrove forests.

2. Statistical Modeling and Scale Separation
The use of a generalized additive mixed model (GAMM) with only 49 data points and three predictors raises concerns about statistical robustness. While relative elevation is a significant driver, the inclusion of non-significant variables (e.g., site area, time since restoration) and the separation of analyses into regional (TSM), site (GAMM), and sub-site (creek network) scales may oversimplify or fragment the interpretation.
Recommendations:
a. Consider simplifying the model or using hierarchical modeling to better integrate multi-scale drivers.
b. Provide more justification for the scale-separated analyses or explore integrated modeling approaches.
c. Discuss the limitations of small sample size and potential overfitting.

3. Global Restoration Potential Mapping
The global projection of restoration potential is a valuable contribution. However, the coarse threshold-based approach may oversimplify complex local conditions. Notably, restoration opportunities in Australia and Africa appear underrepresented (Fig. 3), despite known wetland losses and active restoration programs – reflecting geographic bias in your dataset.
Recommendation: Please clarify the limitations of the threshold approach and discuss how local geomorphic and ecological contexts might affect restoration feasibility. Including regional case studies or sensitivity analyses could enhance the credibility of the global estimates.
Minor Comments
• The manuscript would benefit from a clearer justification for the selection of thresholds (e.g., TSM > 15 g/m^3, elevation > 80%) and a more detailed explanation of how these values were validated.
• Consider elaborating on how the framework could inform practical restoration planning, especially in data-poor regions (such as Africa).

• The figures are informative but could be improved with clearer legends and consistent terminology (e.g., “tidal wetlands” vs. “tidal marshes”).

Reviewer #2

(Remarks to the Author)
General comments
• This manuscript provides a valuable synthesis and develops a ‘cross-scale framework’ of the critical factors affecting the restoration trajectories of managed dyke realignment sites in Europe, notably regional sediment supply, site level relative elevation and sub-site tidal creek connectivity. It goes beyond modelling to link these factors to restoration ‘success’, measured as increased tidal wetland vegetation extent and tidal wetland area over time.
• The work is novel, conclusions are supported by well-designed methods and analyses and the paper will make a valuable contribution to the literature.
• The manuscript is very well written and accessible to a broader audience.
• The link to the restoration benefits of tidal flat ecosystems is less solid (likely associated with fewer publications that quantitatively provide evidence of their contribution) although areas are quantified in this paper.
• It would be beneficial within the discussion to acknowledge and differentiate the overall resilience of minerogenic marshes (which appears to be most of those used in this study) from organogenic marshes, this would strengthen the overall paper and expand its applicability (e.g group sites used in the analysis into these two categories)
• The paper is sensitive to the sites that are reported in the 2021 ABPmer database which is not exhaustive and does not include many of the sites that are reported in Canada or USA for example despite these studies being published. The study rightly focuses on MR within Europe and this is clearly indicated however in the discussion would be beneficial more explicitly speak to the global ‘representativeness’ of the European site relative to tidal wetlands found elsewhere. Minerogenic marshes for example have different tidal creek channel densities compared with micro or meso tidal organogenic systems.
• One of the novel elements of this paper is its effective linking of science with concrete recommendations and practical insights for practitioners (L. 304-309).
• What the limitations of the study? And assumptions made?
Specific Comments
• L. 78 – Esteves 2014 does not appear in the reference list
• L.79 – AMPimer, 2021 – date does not appear in the reference
• L.120 – units for marsh change rate > 0
• L. 121-125 – “Currently most sites are dominated by marsh vegetation, whereas a smaller subset remains predominantly tidal flats, highlighting that both vegetated and unvegetated tidal wetlands represent meaningful restoration outcomes depending on site conditions” – this paper doesn’t provide evidence of the benefits that unvegetated tidal flats represent therefore this statement is an overstatement. It does calculate area but there is not evidence to support the connection with restoration success.
• L368 – “Given that 90% of the documented MR project globally are located in Europe” – this statement is misleading. It is correct that 90% of projects described in the OMREG database are located in Europe but OMREG is a voluntary repository and was not designed to be the definitive representation of project globally. It has also grown significantly in the last few years as awareness of the platform has grown.
• L.402 – assuming that the high resolution Google Earth Imagery used for validation was used from the same year. This should be indicated.
• L476 – confirm that for calculation of mean elevation this excludes elevations within tidal creeks which would have been in the DTM and focuses only on the marsh platform.
• L521 – Maarten, 2024 not in ref list
• L.534 – “we defined a minimum threshold of 15 g/m3 (15 mg/L) to screen areas with adequate sediment supply. This value is consistent with the global analysis by Grandjean et al., 2024…” was this value selected because of that global analysis (which would then be ‘based on” rather than ‘consistent with’) or was other analyses conducted and therefore this is a comparison. What about other papers such as Kirwan et al., 2010 (https://agupubs.onlinelibrary.wiley.com/doi/10.1029/2010gl045489) that states that 20 mg/L is the threshold for SSC for marsh survival. Was this where the upper bound for the sensitivity testing (L544) came from? If so, should be referenced. Providing the SSC used in the analysis for each site in the supplementary materials would be a strong contribution.
• L541 – referencing Kirwan et al., 2016 Overestimation of marsh vulnerability to sea level rise Nature Communications) fig 3 for example for the statement regarding acknowledgement of variation with tidal range and geomorphic setting.
• L544 – where are the
• Figure 3 – query lack of sites in Europe, eastern USA and Canada

Version 1:

Reviewer comments:

Reviewer #1

(Remarks to the Author)
I congratulate the authors the detailed and thoughtful revisions. The manuscript has improved substantially, particularly in the expanded geographic scope, the clarification of ecosystem boundaries, the strengthened statistical analyses, and the transparent documentation of global threshold methods. I have only a few remaining suggestions (minor) that would further enhance clarity, robustness, and reproducibility before final acceptance.

1. Lines ~ 48–58: “hydro geomorphological” → “hydro-geomorphological” (hyphenate consistently). “over 80% successfully maintained or expanded wetlands” → consider “>80% of sites maintained or expanded wetlands”;
2. Lines ~78–80: “National and Atmospheric 2024” should be “National Oceanic and Atmospheric Administration (NOAA), 2024”;
3. Units and symbols: Keep % yr^{-1} and g m^{-3} consistently; currently a mix of % per year and % yr^{-1};
4. Line 279: “wetland is expanding” → “wetlands are expanding”;
5. Line 437: “organgenic marshes” → “organogenic marshes”;
6. Line ~ 799-802: Where you explain 73% (optimistic) and 104% (conservative), include one sentence that rationalizes 104% (>MHWS) as a conservative high-cover bound due to rare inundation/occasional storm/HAT events. It’s there, but one consolidating sentence up front would reduce confusion;
7. Use either TSM or SSC but don’t mix; you use TSM consistently, which is fine—ensure units are consistently formatted as g m^{-3} (not “g m^3” or “g/m3”, checking through both main text and supplementary materials);
8. Database citations (ABPmer/OMREG, NOAA, ACRN) include URL + access date (ABPmer looks correct; NOAA needs fix; ACRN is cited as Purandare et al. 2024 and in the Methods—ensure consistency);
9. Regarding statistical modelling
a. Beta regression treatment of 0/1 values: The site scale vegetation cover model uses a beta distribution, which excludes exact 0 or 1 values. The method does not specify how these cases were handled. Please clarify whether: you applied a transformation into (0,1) (e.g., Smithson–Verkuilen adjustment), or used a zero/one inflated beta variant. Confirming this will avoid potential misinterpretation of the model’s assumptions.
b. Proportional odds assumption in the ordinal logistic model: The regional scale analysis uses an ordinal logistic regression. This model assumes proportional odds, but the manuscript does not report whether this assumption was checked. Please report the outcome of a proportional odds check (e.g., Brant test) or confirm robustness under a multinomial or partial proportional odds specification.
c. Joint hierarchical model: The missing indicator approach used in the paper is practical but can introduce bias if missingness is not random. MCMCglmm results also depend on prior choices and convergence diagnostics. Please include a short note in SI summarizing convergence diagnostics (e.g., trace plots, effective sample sizes);
d. Sub site creek efficiency analysis (n = 18): Given the small sample size, results may be sensitive to influential observations. Suggestion: Add a one sentence note that you evaluated leverage or leave one out sensitivity, or include a partial residual plot in the SI.

Reviewer #2

(Remarks to the Author)
In reviewing the revisions provided to "Hydro-geomorphological drivers across scales shape the trajectory of coastal wetland restoration" - I am pleased to say that the authors have addressed all of my previous questions and concerns. The revised manuscript has significantly improved transparency , reliability and statistical robustness. It will be a significant contribution to the literature. The authors 'response to reviewers' was excellent, and very clearly outlined the changes made to the document. I have no reservations for the paper to be published in its current form.

Open Access This Peer Review File is licensed under a Creative Commons Attribution 4.0 International License, which permits use, sharing, adaptation, distribution and reproduction in any medium or format, as long as you give appropriate credit to the original author(s) and the source, provide a link to the Creative Commons license, and indicate if changes were

made.
In cases where reviewers are anonymous, credit should be given to 'Anonymous Referee' and the source.
The images or other third party material in this Peer Review File are included in the article's Creative Commons license, unless indicated otherwise in a credit line to the material. If material is not included in the article's Creative Commons license and your intended use is not permitted by statutory regulation or exceeds the permitted use, you will need to obtain permission directly from the copyright holder.
To view a copy of this license, visit https://creativecommons.org/licenses/by/4.0/

Point-by-point response

We address the reviewers' comments below. Reviewer comments are reproduced verbatim in italics, followed by our responses in regular text. All revisions are shown in the tracked-changes manuscript, with key changes cross-referenced by line number (and by figure/table or Supplementary item where relevant).

Reviewer #1:

Dear Authors,
Thank you for the opportunity to review your manuscript titled "Hydro-geomorphological drivers across scales shape the trajectory of coastal wetland restoration." Your work presents a valuable multi-scale framework for assessing restoration success in managed realignment (MR) projects and contributes meaningfully to the global discourse on Nature-based Solutions (NbS).After careful consideration, I offer the following comments and suggestions to strengthen the manuscript:

Reply: We thank Reviewe#1 for the careful reading and the positive assessment of the manuscript's contribution to multi-scale evaluation of managed realignment and Nature-based Solutions. We also appreciate the constructive suggestions, which helped us substantially strengthen the geographic/ecosystem scope, the statistical robustness, and the interpretation of the global screening maps. Below we address each comment in turn and indicate where changes have been made in the revised manuscript.

Major Comments
1. Geographic and Ecosystem Scope
While the manuscript provides a robust analysis of 49 MR sites in Europe, the exclusive reliance on the OMREG database limits the geographic scope. The Restoration efforts in North America, Australia, and Africa are notably absent, despite their relevance to global restoration targets. The statement that "over 90% of documented MR projects globally are located in Europe" is not quite right as there are many cases available in Northern America (e.g., NOAA) and Australia (Geoscience Australia).
Additionally, mangrove systems—critical coastal wetlands in tropical regions—are not considered, which restricts the ecological applicability of the framework.
Recommendation: A comprehensive literature search would improve the credibility of the research. In particular, any study on coastal wetland much include mangrove forests.

REPLY: We thank the reviewer for this constructive comment and for highlighting the importance of geographic coverage and ecosystem representativeness in studies of coastal wetland restoration. In response, we made three concrete changes: (i) we corrected the wording to avoid overstating Europe's global representativeness and explicitly acknowledge uneven documentation across inventories; (ii) we expanded the empirical basis by compiling additional projects from NOAA Restoration Atlas and ACRN, increasing the dataset from 49 to 69 sites spanning Europe, North America, and Oceania; and (iii) we clarified ecosystem scope by focusing analyses on non-mangrove tidal wetlands (tidal flats and salt marshes), with harmonized

inclusion criteria now detailed in the Methods and Supplementary Materials, and we added a Discussion paragraph on how (and why) the framework may or may not transfer to mangrove systems.

We agree that our original wording overstated the global representativeness of European managed realignment (MR) projects and that substantial restoration efforts exist outside Europe, particularly in North America and Australia. In the revised manuscript, we removed this statement and instead acknowledge that MR projects are unevenly documented across available inventories (see lines 77-80).

To directly address this concern and broaden the empirical basis of our analysis beyond Europe, We conducted a targeted search and compilation of additional restoration data from authoritative databases outside Europe. Specifically, we incorporated information from the U.S. NOAA Restoration Atlas (https://www.fisheries.noaa.gov/resource/map/restoration-atlas) and from the Database for Marine and Coastal Restoration Projects in Australia and New Zealand developed by the Australian Coastal Restoration Network (ACRN, https://www.acrn.org.au/). These sources document numerous coastal restoration projects involving tidal reconnection, dyke removal, and wetland creation, and are now explicitly acknowledged and referenced in the revised manuscript. This expanded compilation increases the analyzed dataset from **49** to **69** sites spanning Europe, North America, and Oceania (see lines 113-114).

To ensure that sites drawn from OMREG, NOAA, and ACRN remain comparable despite differences in reporting formats, we applied harmonized and clearly defined inclusion criteria. Specifically, we focused on projects that (i) targeted tidal wetland ecosystems, explicitly including tidal flats and tidal marshes; (ii) involved restoration actions centred on dyke removal, managed realignment, or the re-establishment of tidal exchange (e.g., managed realignment, dike removal, creation of intertidal wetlands); and (iii) exceeded a minimum restored area of 10 ha, ensuring comparability with large-scale MR projects analysed in Europe. These screening criteria and the resulting dataset are now described more explicitly in the Methods and Supplementary Materials (see lines 481-508; Table S7).

Regarding ecosystem scope, we clarify that our quantitative analyses focus on non-mangrove tidal wetlands (tidal flats and salt marshes) (see lines 80-82). Projects primarily focused on mangrove restoration were excluded deliberately for two reasons. First, mangrove ecosystems are characterized by woody vegetation

and distinct sedimentary, hydrodynamic, and creek-network feedbacks that differ fundamentally from those of tidal flats and salt marshes. Second, mangrove-based managed realignment projects remain sparsely represented across the three databases we compiled (OMREG, NOAA, and ACRN), limiting their suitability for a robust and comparable cross-site analysis at present (see lines 490-498). Nevertheless, we recognize the global importance of mangrove ecosystems and now explicitly discuss their relevance, key differences, and the potential applicability and limitations of our framework for mangrove systems in the Discussion, citing representative examples from the literature (see lines 382-399).

Together, we hope these revisions improve the transparency and interpretability of the manuscript with respect to ecosystem scope.

2. Statistical Modeling and Scale Separation

The use of a generalized additive mixed model (GAMM) with only 49 data points and three predictors raises concerns about statistical robustness. While relative elevation is a significant driver, the inclusion of non-significant variables (e.g., site area, time since restoration) and the separation of analyses into regional (TSM), site (GAMM), and sub-site (creek network) scales may oversimplify or fragment the interpretation.

Recommendations:

a. Consider simplifying the model or using hierarchical modeling to better integrate multi-scale drivers.

b. Provide more justification for the scale-separated analyses or explore integrated modeling approaches.

c. Discuss the limitations of small sample size and potential overfitting.

REPLY: We thank the reviewer for the detailed comments on statistical robustness, scale separation, and potential overfitting. In response, we made four concrete revisions. First, we simplified the site-scale vegetation-cover analysis by implementing explicit model selection and refitting a parsimonious penalized beta-GAM, and we added repeated random-split cross-validation (80% training, 20% testing; 10 repeats) plus nonparametric bootstrap refitting (B = 500) to evaluate predictive stability and quantify uncertainty (see lines 624-646; Fig. S8). Second, we strengthened the regional-scale sediment analysis by complementing the nonparametric comparisons with an ordinal logistic regression linking TSM to an ordered loss–stable–gain gradient while testing tidal range as a covariate (see lines 591-602; Table S1). Third, to integrate multi-scale drivers within a single statistical framework, we introduced a joint multivariate hierarchical model that links each recovery dimension to its scale-aligned driver and tests whether outcomes are statistically coupled across sites (see lines 678-737; Tables S2–S4). Finally, we

expanded the Discussion to explicitly acknowledge limitations related to sample size, uneven data availability among response variables, and potential overfitting, and to justify the ecological rationale for analyzing drivers at their operative scales while interpreting them within a unified cross-scale framework (see lines 325-342; 423-427).

First, to address concerns about model complexity and non-informative predictors at the site scale, we implemented explicit model selection and simplification for the vegetation-cover analysis. Using multi-model comparison, we found that restoration area and time since restoration did not improve model support once relative elevation was included, and the best-supported model retained relative elevation as the sole predictor (see lines 604-646). We therefore refitted a parsimonious beta GAM with a penalized spline for relative elevation. To evaluate predictive stability and guard against overfitting, we assessed performance using repeated random-split cross-validation (80% training / 20% testing; 10 repeats) and summarized prediction error using RMSE (median RMSE = 0.213 on the 0–1 scale; 10.7% improvement over a mean-only reference). We further conducted bootstrap refitting (B = 500) and found the elevation smooth remained significant in 99.8% of refits, and we report the corresponding uncertainty band in Fig. S8.

Second, for the regional-scale sediment analysis, we retained the non-parametric comparison of TSM across wetland change categories but strengthened inference by adding an ordinal logistic regression to quantify how sediment supply shifts the probability of sites transitioning along a loss–stable–gain gradient, while also testing tidal range as a hydrodynamic covariate (see lines 591-602). This regression-based analysis yielded consistent results, with higher TSM significantly increasing the odds of more positive restoration trajectories (P = 0.004), while tidal range was not significant (P = 0.31; see lines 151-155). Importantly, this modelling framework provides a consistent statistical basis for the subsequent threshold analysis by linking sediment supply to fitted transition probabilities (see lines 599-602).

Third, to address the concern that scale-separated analyses may fragment interpretation, we added a joint hierarchical modelling framework to integrate evidence across regional-, site-, and sub-site-scale drivers within a single model structure (see lines 117-123; 678-737). The joint model simultaneously analyses three restoration dimensions (tidal wetland non-loss, current vegetation cover, and early-stage colonization rate), includes an unstructured site-level covariance structure to test whether these outcomes are statistically coupled across sites, and

links each response to its primary scale-aligned driver (TSM, relative elevation, and creek density, respectively). The joint model identified the same scale-aligned relationships and showed that these recovery dimensions did not consistently improve together across sites (see lines 181-188). As a robustness check, adding secondary hydro-geomorphological covariates to each response did not materially change inference on the primary effects (see lines 188-189, 722-732).

Finally, we now explicitly discuss limitations related to sample size, uneven data availability across response variables, and potential overfitting. Although the revised dataset expands to 69 sites, it remains insufficient to support highly parameterized or fully process-based modelling at the global scale, particularly for sub-site processes where tidal creek analyses rely on a smaller subset of sites (see lines 423-427). Accordingly, we emphasize parsimonious, physically interpretable predictors and interpret results within this screening-level scope (see lines 427-437). We also expand the Discussion to explain the ecological rationale for scale separation. Specifically, we show that sediment supply, relative elevation, and tidal creek networks act as distinct constraints operating at different spatial scales, and we clarify how integrating these drivers within a cross-scale framework improves interpretability rather than fragmenting inference (see lines 325-342).

3. Global Restoration Potential Mapping

The global projection of restoration potential is a valuable contribution. However, the coarse threshold-based approach may oversimplify complex local conditions. Notably, restoration opportunities in Australia and Africa appear underrepresented (Fig. 3), despite known wetland losses and active restoration programs – reflecting geographic bias in your dataset. Recommendation: Please clarify the limitations of the threshold approach and discuss how local geomorphic and ecological contexts might affect restoration feasibility. Including regional case studies or sensitivity analyses could enhance the credibility of the global estimates.

REPLY: We appreciate the reviewer's positive assessment of the global projection and agree that a threshold-based screening inevitably simplifies site specific geomorphic and ecological constraints. In response, we made four concrete revisions. First, we redefined the TSM and relative elevation thresholds using a model informed workflow and benchmarked them against published evidence as an external plausibility check (see lines 772-805; Table S10; Table S11). Second, we updated the global stepwise screening using the revised central thresholds (TSM = 20.4 g m^{-3}; relative elevation = 90.6% of the local tidal frame) and explicitly propagated threshold uncertainty through both scenario based sensitivity analysis and Monte Carlo simulations (see lines 806-858; Table S5; Figure S4). Third, we now interpret the global maps explicitly as a conservative,

screening level assessment of physically plausible restoration space rather than site specific predictions, and we expand discussion of local context dependence and dynamic elevation change that can shift realised outcomes through time (see lines 409-445). Fourth, we clarify that apparent regional gaps in Australia and Africa can arise from the loss based candidate pool and ecosystem composition rather than absence of restoration opportunity, and we discuss how wave dominated or mixed energy settings and mangrove dominated coastlines can modulate feasibility and lead to conservative underestimation in the screening maps (see lines 446-465).

(1) Threshold definition and external benchmarking

We derived the primary sediment threshold from the fitted loss no change gain ordinal model as the gain 50 threshold, namely the TSM level where the predicted probability of wetland gain reaches 0.5. This occurred at 20.4 g m^{-3}. Uncertainty was quantified using nonparametric bootstrap resampling, yielding a 95% interval of 14.47 to 26.42 g m^{-3}, which we carry forward into the global analyses (see lines 772-787; Table S10). In parallel, we derived the operational relative elevation threshold from the fitted elevation cover relationship using a functional establishment criterion (predicted cover ≥ 0.8). The central threshold was 90.6% of the local tidal frame, with an uncertainty envelope of 73 to 104% based on prediction uncertainty bounds (see lines 788-805; Table S11; Fig. S9).

(2) Sensitivity analyses and uncertainty propagation

We implemented two complementary sensitivity components. A discrete scenario analysis combines low central high sediment thresholds with optimistic central conservative elevation thresholds to form nine screening scenarios, which bracket global estimates (see lines 806-842; Table S5). In addition, we propagated uncertainty using Monte Carlo simulations by sampling sediment thresholds across 14.47 to 26.42 g m^{-3} and elevation thresholds across 73 to 104% and repeating the screening at the grid cell level ($n = 100$; see lines 843-858). The resulting spatial uncertainty maps report SD, CV, and 95% interval width (see Figure S4), and show that cell level uncertainty is generally moderate (mean SD = 0.17 km^2 for sediment screened potential and 0.09 km^2 for vegetation eligible potential; see lines 203-208; Figure S4).

(3) Interpretation and local context dependence

We now emphasize that the global maps provide a first order, physically based screening rather than site specific predictions. Even where thresholds are met, realised feasibility can vary with wave exposure, substrate composition, salinity

regimes, propagule availability, and project design (see lines 409-421). We also explicitly note that elevation is dynamic after reconnection and that accretion or erosion can shift the effective vegetation window through time, which is not resolved in a static screening framework (see lines 438-445).

(4) Apparent regional gaps and geographic bias

We clarify that underrepresentation in Australia and Africa can reflect limited mapped direct anthropogenic tidal wetland loss during 1999 to 2019 in the candidate loss dataset, mangrove dominated coastlines outside our non-mangrove scope, and conservative thresholds applied to wave dominated or mixed energy settings with low to moderate suspended sediment availability (see lines 446-465; Fig. 3). We therefore present Fig. 3 as a conservative, screening level assessment, and note that regional hydro-geomorphological regimes and ecosystem composition can modulate realised feasibility (see lines 462-465).

Minor Comments

• The manuscript would benefit from a clearer justification for the selection of thresholds (e.g., TSM > 15 g/m³, elevation > 80%) and a more detailed explanation of how these values were validated.

REPLY: We agree that the manuscript benefits from a clearer justification and documentation of the screening thresholds. In the revision, we replaced the previously stated illustrative cutoffs (e.g., TSM > 15 g m^{-3}; elevation > 80%) with a transparent three-step workflow that specifies (i) how thresholds were derived, (ii) how uncertainty was quantified and propagated, and (iii) how the resulting values were benchmarked against published evidence as an external plausibility check.

(1) How the thresholds were derived (model-informed definitions).

For sediment supply, we used the fitted loss–no change–gain ordinal model to predict outcome probabilities across the observed TSM gradient, with tidal range fixed at its median value to provide a representative hydrodynamic context. We then defined the primary sediment screening threshold as the gain-50 threshold, namely the TSM level at which the predicted probability of tidal wetland gain reaches 0.5. This occurred at 20.4 g m^{-3} (see lines 772-787).

For relative elevation, we used the fitted elevation–cover relationship and defined a functional establishment criterion (predicted vegetation cover ≥ 0.8). The primary elevation threshold was identified as the lowest relative elevation at which the fitted mean prediction exceeded this criterion, corresponding to 90.6% of the local tidal frame (see lines 788-805; Figure S9).

(2) How uncertainty was quantified and propagated .

We quantified uncertainty in the sediment threshold using bootstrap resampling, yielding a 95% interval of 14.9–26.9 g/m³, and characterized uncertainty in the elevation threshold using prediction uncertainty bounds, yielding an envelope of 80.7–101.3% of the local tidal frame (see lines 772-805). To evaluate how sensitive the global estimates are to plausible threshold uncertainty, we propagated these ranges through the global screening using a two-level sensitivity analysis: (i) nine discrete scenarios formed by factorial combinations of low/central/high TSM thresholds and optimistic/central/conservative elevation thresholds (3×3; Table S5), and (ii) Monte Carlo uncertainty propagation by sampling thresholds continuously within these envelopes and repeating the grid-cell screening procedure (n = 100) (see lines 806-858). We now report spatially explicit

uncertainty metrics and show that grid-cell–level uncertainty was generally moderate across simulations (Figure S4; see lines 203-208).

(3) How thresholds were benchmarked against external evidence (plausibility checks).

Finally, we benchmarked the model-derived thresholds against published empirical and process-based evidence as an external plausibility check. The sediment threshold (20.4 g/m^3) aligns closely with reported sediment requirements for tidal wetland persistence (~20 g/m^3; Table S10). The elevation threshold (90.6% of the local tidal frame) is consistent with empirical and mechanistic expectations that persistent marsh establishment occurs toward the upper tidal frame, as supported by prior studies synthesised in Table S11 (see lines 771-805; Table S10; Table S11). These benchmarks are summarized in the new Supplementary Tables S10 (sediment thresholds) and S11 (relative-elevation evidence), which compile published ranges and mechanistic/empirical support alongside our model-derived values.

• *Consider elaborating on how the framework could inform practical restoration planning, especially in data-poor regions (such as Africa).*

REPLY: We agree and have expanded the Discussion to clarify how the framework can inform practical restoration planning, particularly in data-poor regions (e.g., parts of Africa). We now present the framework as a stepwise decision-support tool that can be applied even when long-term monitoring data and comprehensive restoration inventories are lacking, because it relies on a small number of physically interpretable variables that are globally available from remote sensing and modelling products (see lines 344-399).

First, at the screening and prioritization stage, the framework can be used to identify historically lost tidal wetlands that remain physically restorable under current sediment availability, helping narrow a large search space to a smaller set of plausible opportunity areas where limited resources for field surveys and stakeholder engagement can be targeted most efficiently (see lines 357-371). Where relevant, these screened opportunities can also be benchmarked against the Kunming–Montreal Global Biodiversity Framework 30% restoration target to support strategic planning and reporting.

Second, once candidate areas are identified, the framework provides actionable design guidance by linking each dominant constraint to practical interventions. We

highlight that sediment pathways and budgets should be considered early in planning, thin-layer sediment placement can be used to raise platform elevation where marsh establishment is desired, and initiating creek networks that mimic natural patterns can improve drainage and facilitate early colonization (see lines 345-356). We also note that the elevation-based component helps differentiate likely trajectories toward vegetated tidal marshes versus persistent tidal flats, which can help align restoration objectives with local ecological priorities (see 366-371).

Finally, we emphasize that in data-poor settings the framework is not intended to replace detailed local feasibility assessments, but rather to provide a transparent, first-order basis for triaging opportunities and sequencing follow-up work, thereby improving the efficiency and robustness of restoration planning under limited data and capacity (see lines 372-381).

• *The figures are informative but could be improved with clearer legends and consistent terminology (e.g., "tidal wetlands" vs. "tidal marshes").*

Reply: We thank the reviewer for this helpful suggestion. We have revised the figure legends and captions to improve readability and to ensure consistent terminology throughout. Specifically, we now use "tidal wetlands" to refer to the combined intertidal habitat space (including tidal flats and vegetated tidal marshes), whereas "tidal marsh" is used only when referring explicitly to vegetated marsh habitat or marsh vegetation cover. We checked all figures, captions, and axis labels to align with these definitions. In addition, we clarified legends by expanding abbreviations, adding units and threshold annotations where relevant, and ensuring that color scales and category labels (e.g., loss/no change/gain) are defined consistently across figures.

Reviewer #2:

General comments

• This manuscript provides a valuable synthesis and develops a 'cross-scale framework' of the critical factors affecting the restoration trajectories of managed dyke realignment sites in Europe, notably regional sediment supply, site level relative elevation and sub-site tidal creek connectivity. It goes beyond modelling to link these factors to restoration 'success', measured as increased tidal wetland vegetation extent and tidal wetland area over time.

• The work is novel, conclusions are supported by well-designed methods and analyses and the paper will make a valuable contribution to the literature.

• The manuscript is very well written and accessible to a broader audience.

> **Reply**: We thank Reviewer #2 for these encouraging and constructive general comments. We are pleased that the reviewer finds the manuscript novel, well supported by the analyses, and accessible to a broad audience. We also appreciate the reviewer's recognition of the cross-scale framework and our effort to link regional sediment supply, site-scale relative elevation, and sub-site tidal creek connectivity to measurable restoration trajectories and indicators of restoration performance. We have carefully considered the reviewer's detailed suggestions and have revised the manuscript accordingly, which we believe further strengthens the clarity, robustness, and practical relevance of the study.

• The link to the restoration benefits of tidal flat ecosystems is less solid (likely associated with fewer publications that quantitatively provide evidence of their contribution) although areas are quantified in this paper.

> **Reply:** We thank the reviewer for this thoughtful point and agree that quantitative evidence linking restoration success to ecosystem service provision is currently more extensive for vegetated tidal marshes than for unvegetated tidal flats, reflecting a historical emphasis on plant-mediated functions (see lines 268-270). We have therefore revised the Discussion to frame tidal-flat values more clearly and cautiously. Specifically, we now acknowledge the relative scarcity of service-based quantitative evidence for tidal flats compared with marshes, while emphasizing that tidal flats nonetheless provide well-recognized ecological functions, including shorebird foraging habitat, benthic food-web support, and contributions to sediment dynamics (see lines 271-274). Importantly, we clarify that, in our framework, the persistence of tidal flats following managed realignment should not automatically be interpreted as restoration failure, but rather as an alternative and potentially stable outcome depending on local physical conditions and management objectives (see lines 274-276). Consistent with this

framing, we also highlight that our global screening distinguishes areas more likely to recover as vegetated tidal marshes versus those more likely to persist as tidal flats, enabling practitioners to tailor restoration goals to local ecological needs (e.g., maximizing vegetation establishment versus maintaining critical foraging habitat for shorebirds) (see lines 366-371).

• *It would be beneficial within the discussion to acknowledge and differentiate the overall resilience of minerogenic marshes (which appears to be most of those used in this study) from organogenic marshes, this would strengthen the overall paper and expand its applicability (e.g group sites used in the analysis into these two categories)*

Reply: We thank the reviewer for this important suggestion and agree that explicitly distinguishing minerogenic versus organogenic marsh contexts strengthens interpretation and broadens applicability. In the revised Discussion, we clarify that the empirical basis of our screening framework is most representative of temperate, minerogenic saltmarsh systems, particularly European managed realignment sites, where elevation change and persistence are strongly influenced by external mineral sediment supply (see lines 422-432). We further note that the dominant accretion pathways and thus overall resilience can differ in organogenic marshes, where in situ organic production and biogenic feedbacks may play a larger role and creek-network characteristics may differ, which can limit direct transferability of thresholds inferred from minerogenic settings (Kirwan & Megonigal 2013) (see lines 433-437).

With respect to the reviewer's helpful suggestion to group sites into minerogenic versus organogenic categories, we agree this would be valuable. However, implementing such a classification robustly at the global scale would require consistent, site-specific indicators of accretion sources or organic production (e.g., partitioning mineral versus organic contributions, or comparable productivity/peat-formation metrics), which are not systematically available across the managed realignment sites compiled here. We therefore did not apply an explicit two-group classification in the current analysis, and we caution that thresholds and resilience patterns inferred largely from minerogenic settings should not be directly extrapolated to organogenic marshes without additional local evidence (see lines 435-437).

• *The paper is sensitive to the sites that are reported in the 2021 ABPmer database which is not exhaustive and does not include many of the sites that are reported in Canada or USA for example despite these studies being published. The study rightly focuses on MR within Europe and this is clearly indicated however in the discussion would be beneficial more explicitly*

speak to the global 'representativeness' of the European site relative to tidal wetlands found elsewhere. Minerogenic marshes for example have different tidal creek channel densities compared with micro or meso tidal organogenic systems.

Reply: We agree that the ABPmer/OMREG database is not exhaustive and that relying on it alone can bias inference by under-representing MR-type projects reported elsewhere. In response, we (i) removed the wording that implied global representativeness and now explicitly acknowledge uneven documentation across inventories; (ii) expanded the empirical basis by compiling additional projects from the NOAA Restoration Atlas and the Australian Coastal Restoration Network (ACRN), increasing the dataset from 49 to 69 sites; (iii) applied harmonised inclusion criteria to ensure comparability across OMREG, NOAA and ACRN and summarised the screening workflow in a dedicated flow diagram; and (iv) added a clearer Discussion of representativeness and transferability limits of the predominantly European evidence base.

We agree that the 2021 ABPmer/OMREG database is not exhaustive and that relying on it alone could under-represent MR-type projects reported elsewhere (e.g., Canada and the USA). We therefore removed wording that implied global representativeness and now explicitly acknowledge that MR projects are unevenly documented across available inventories (see lines 75-80).

To reduce sensitivity to any single inventory and broaden the empirical basis beyond Europe, we compiled additional MR-type projects from the NOAA Restoration Atlas and the Australian Coastal Restoration Network (ACRN), expanding the analysed dataset from 49 to 69 sites spanning Europe, North America, and Oceania (see lines 480-508; Table S7).

To ensure that sites drawn from OMREG, NOAA, and ACRN remain comparable despite differences in reporting formats, we applied harmonised inclusion criteria (tidal wetland targets including tidal flats and salt marshes; dyke realignment/tidal reconnection interventions; restored area > 10 ha). We also summarise the stepwise screening workflow in a dedicated flow diagram (see lines 499-508; Fig. S5).

Finally, we now more explicitly discuss the global representativeness and transferability limits of the predominantly European evidence base. Specifically, we note that the dataset is most representative of temperate, minerogenic saltmarsh systems, and that transferability to organogenic marshes may be limited because

in situ organic production and biogenic feedback can play a larger role and creek-network properties may differ, potentially affecting sub-site connectivity controls (see lines 422-437).

• *One of the novel elements of this paper is its effective linking of science with concrete recommendations and practical insights for practitioners (L. 304-309).*

Reply: We thank the reviewer for highlighting this practitioner-oriented contribution. Building on the existing "Towards optimized Nature-based restoration in coastal zones" section (L. 304–309), we further strengthened the Discussion to make the management implications more concrete and explicitly tied to the three hydro-geomorphological constraints identified in our analyses. Specifically, we now recommend that practitioners (i) evaluate sediment transport pathways and budgets early in project planning, (ii) consider thin-layer sediment placement to raise platform elevation where marsh establishment is desired, and (iii) initiate or design tidal creek networks that mimic natural patterns to improve drainage and accelerate early colonization in low-lying sites (see lines 345-356).

We also clarify how these site-level insights can be scaled up for strategic planning. In the revised Discussion, we describe how the global screening can be used to prioritize managed realignment opportunities under current sediment availability and to distinguish areas more likely to develop as vegetated marshes versus persist as tidal flats, thereby supporting goal-setting aligned with local ecological objectives (e.g., vegetation recovery versus shorebird foraging habitat) (see lines 357-371). Finally, we explicitly note that this parsimonious framework, based on a small set of physically interpretable variables available from remote sensing and tidal models, is particularly useful as a first-order screening tool in data-poor regions (including parts of Africa), helping target where limited resources for field surveys, pilot projects, and stakeholder engagement can be most effectively deployed rather than replacing detailed local feasibility assessments (see lines 372-381).

• *What the limitations of the study? And assumptions made?*

Reply: We thank the reviewer for requesting a clearer statement of the study's limitations and assumptions. In the revised manuscript, we added a dedicated Discussion subsection ("Uncertainties and interpretation of global restoration estimates") to explicitly summarise the main constraints on interpretation,

transferability, and the scope of inference (see lines 400-477). We also clarify the key assumptions underlying the framework (see lines 102-110).

Limitations

First, our global restoration estimates are produced by a parsimonious, screening level framework that provides a conservative, process-based screening of physically plausible recovery space rather than site specific predictions. We explicitly vary sediment and elevation thresholds to define a sensitivity envelope, but even where these thresholds are met, local geomorphic and ecological conditions, including wave exposure, substrate composition, salinity regimes, propagule availability, and project design, can further constrain feasibility and influence realised outcomes (see lines 417-4211).

Second, sediment requirements for wetland maintenance and recovery are not universal and may vary with tidal range and hydrodynamic regime. As a result, the framework may still overestimate restoration potential in macrotidal environments and underestimate it in microtidal settings, despite using relative elevation metrics (see lines 409-416).

Third, beyond parameter uncertainty, transferability is constrained by data coverage and representativeness. Although the number of managed realignment sites analysed here is larger than in most previous remote sensing based evaluations, it remains insufficient to support highly parameterised or fully process based modelling at the global scale, particularly for sub site processes where tidal creek analyses rely on a smaller subset of sites. Empirically, the evidence base is most representative of temperate, minerogenic saltmarsh systems, and transferability to organogenic marshes, where in situ organic production and biogenic feedbacks can play a larger role and creek network properties may differ, may therefore be limited (see lines 422-437).

Fourth, wetland elevation is not static following managed realignment. Restored platforms may accrete or erode through time, shifting relative elevation and altering the elevation window suitable for marsh establishment. These dynamic feedbacks between sediment supply, morphology, and vegetation are not explicitly resolved in the screening framework (see lines 438-445).

Finally, we now state explicitly that the global maps represent a conservative, process-based screening of upper-bound physical potential rather than a comprehensive inventory of restoration pathways. We also explain that low

mapped potential can arise for two distinct reasons: (i) the candidate pool is defined by direct anthropogenic tidal-wetland loss during 1999–2019, so regions with sparse eligible loss in this time window (e.g., much of Europe, the eastern USA, and Canada) can show limited potential mainly in absolute terms; and (ii) where losses do occur, only a subset meets the hydro-geomorphological thresholds used in the screening. In addition, we note that parts of Oceania and Africa may be conservatively underestimated because mapped losses are relatively limited and many coastlines are mangrove-dominated (outside our non-mangrove scope), and because wave-dominated or mixed-energy estuaries with lower suspended sediment availability may be screened out under conservative sediment thresholds. We therefore emphasize that the maps should be complemented by site-specific assessments that incorporate local geomorphic context, ecosystem type, and management objectives (see lines 446–465).

Assumptions

Conceptually, the framework is built on the hypothesis that, although many contextual factors can influence restoration outcomes, fundamental hydro geomorphological constraints operating across spatial scales will emerge as dominant and comparatively transferable drivers when evaluated within a unified framework. Specifically, we hypothesised that regional scale sediment supply, site scale relative elevation, and sub site drainage structure would consistently explain variation in restoration outcomes across managed realignment sites, even after accounting for other commonly cited factors such as tidal range, restoration area, and restoration duration (see lines 102-110).

Specific Comments

• *L. 78 – Esteves 2014 does not appear in the reference list*

• *L.79 – AMPimer, 2021 – date does not appear in the reference*

Reply: Thank you for noting these reference issues. We have corrected and updated the reference list accordingly. (i) The citation to Esteves (2014) has now been added to the reference list. (ii) For ABPmer/OMReg, which is an online database rather than a dated journal publication, we have revised the entry to include the URL and an explicit access date, and we have updated the in-text citation accordingly (e.g., “ABPmer, accessed 2026”), avoiding an unsupported publication year. We also re-checked the manuscript to ensure all in-text citations are included in the reference list with consistent authorship and dating.

• *L.120 – units for marsh change rate > 0*

Reply: Thank you for noting this. We have now added the units for the tidal marsh change rate (and in the corresponding figure/legend where relevant) to clarify that this rate is expressed as % per year (see lines 133). We also reviewed the manuscript to ensure that units are consistently reported for all key variables and response metrics throughout the main text, figures, and tables.

• *L. 121-125 – "Currently most sites are dominated by marsh vegetation, whereas a smaller subset remains predominantly tidal flats, highlighting that both vegetated and unvegetated tidal wetlands represent meaningful restoration outcomes depending on site conditions" – this paper doesn't provide evidence of the benefits that unvegetated tidal flats represent therefore this statement is an overstatement. It does calculate area but there is not evidence to support the connection with restoration success.*

Reply: Thank you for noting this. We agree that our original wording at L.121 to 125 could be interpreted as overstating the evidence for treating unvegetated tidal flats as a meaningful restoration outcome in terms of demonstrated benefits. Our analyses quantify post restoration habitat trajectories and area changes, but they do not directly measure ecosystem service delivery or other benefit based indicators. We have therefore removed this statement from the Results and early narrative to avoid implying a benefit based link between tidal flat persistence and restoration success (see lines 135-137).

We have instead revised the Discussion to present a more cautious and appropriately supported framing. We now explicitly acknowledge that quantitative evidence linking restoration outcomes to ecosystem service provision is more extensive for vegetated marshes than for unvegetated tidal flats (see lines 268-271). Where we discuss tidal flats, we cite established literature describing their ecological functions, such as shorebird foraging habitat, benthic food web support, and contributions to sediment dynamics (see lines 271-276). We also reframe tidal flat persistence following managed realignment as an alternative and potentially stable post restoration trajectory that may be desirable depending on local physical conditions and management objectives, and we avoid using it as a universal indicator of success (see lines 366-371).

• *L368 – "Given that 90% of the documented MR project globally are located in Europe" – this statement is misleading. It is correct that 90% of projects described in the OMREG database are located in Europe but OMREG is a voluntary repository and was not designed*

to be the definitive representation of project globally. It has also grown significantly in the last few years as awareness of the platform has grown.

Reply: We thank the reviewer for this important clarification and agree that our original wording at L368 was misleading. Our intent was to describe the coverage of the OMREG database rather than to imply that Europe contains about 90 % of managed realignment projects globally. In the revised manuscript, we removed the "90 % globally" statement and now state that managed realignment is unevenly documented across available inventories (see lines 77-80).

To reduce sensitivity to any single database and to broaden the empirical basis beyond Europe, we conducted a targeted compilation of additional MR-type projects from authoritative sources outside OMREG. Specifically, we incorporated projects from the U.S. NOAA Restoration Atlas and from the Australian Coastal Restoration Network (ACRN) database for Australia and New Zealand. These sources are now explicitly acknowledged and referenced in the revised manuscript, expanding the analysed dataset from 49 to 69 sites spanning Europe, North America, and Oceania (see lines 481-508; Table S7).

To ensure comparability across OMREG, NOAA, and ACRN despite differences in reporting formats, we applied harmonized inclusion criteria focused on (i) tidal wetland targets (tidal flats and tidal marshes), (ii) MR or functionally equivalent tidal reconnection interventions (e.g., dike breaching/removal), and (iii) a minimum restored area of 10 ha. These screening decisions and the resulting dataset are now described more transparently in the Methods and Supplementary Materials (see lines 490-508, Fig. S5).

We hope these revisions clarify the correct interpretation of OMREG coverage statistics, avoid overgeneralizing database proportions to the global managed realignment footprint, and improve transparency regarding database scope and representativeness.

• *L.402 – assuming that the high resolution Google Earth Imagery used for validation was used from the same year. This should be indicated.*

Reply: Thank you for noting this. We have now clarified in the Methods that the high-resolution Google Earth imagery used for validation was not restricted to a single calendar year, because restoration commenced in different years across sites. Instead, for each site we selected a cloud-free, vegetation-season Google Earth

image from the site-specific year corresponding to its reported post-restoration age, so that validation reflects comparable restoration phases across sites. We also state this selection rule explicitly in the revised text (see lines 533-537) and provide the corresponding image years in Table S8.

• *L476 – confirm that for calculation of mean elevation this excludes elevations within tidal creeks which would have been in the DTM and focuses only on the marsh platform.*

Reply: Thank you for this helpful clarification. Mean elevation in our site-scale analysis was calculated as the area-averaged DTM elevation within the mapped restoration boundary (i.e., it was not restricted to the marsh platform only and may include low-elevation tidal creek beds). To evaluate whether this could bias our elevation metric, we conducted a sensitivity check for the subset of sites with mapped tidal creek vectors (n = 18). Excluding a 1-m buffer around mapped creek lines increased mean elevation by only 0.056 m on average (median 0.037 m), indicating that potential contamination of mean elevation by creek pixels is minor. We have added/clarified this point in the Methods (see lines 619-623)

• *L521 – Maarten, 2024 not in ref list*

Reply: Thank you for noting these reference issues. We have corrected and updated the reference list accordingly (see lines 762-763).

• *L.534 – "we defined a minimum threshold of 15 g/m3 (15 mg/L) to screen areas with adequate sediment supply. This value is consistent with the global analysis by Grandjean et al., 2024…" was this value selected because of that global analysis (which would then be 'based on" rather than 'consistent with') or was other analyses conducted and therefore this is a comparison. What about other papers such as Kirwan et al., 2010 (https://agupubs.onlinelibrary.wiley.com/doi/10.1029/2010gl045489) that states that 20 mg/L is the threshold for SSC for marsh survival. Was this where the upper bound for the sensitivity testing (L544) came from? If so, should be referenced. Providing the SSC used in the analysis for each site in the supplementary materials would be a strong contribution.*

Reply: We thank the reviewer for this careful reading. We agree that our previous wording around an illustrative "15 g/m^3 (15 mg/L)" cutoff could be interpreted as unclear regarding whether it was based on Grandjean et al. (2024) or merely compared with it. In the revision, we removed the fixed 15 g/m^3 cutoff and replaced it with a transparent workflow that (i) derives the sediment threshold from our empirical data, (ii) quantifies uncertainty and propagates it through sensitivity analyses, and (iii) benchmarks the resulting values against published evidence as an external plausibility check (see lines 771-858, Table S5 and S10).

(1) Threshold derivation (model-informed, not taken from Grandjean et al. 2024).

For sediment supply, the primary screening threshold was derived from our fitted loss–no change–gain ordinal model by predicting outcome probabilities across the observed TSM gradient (with tidal range fixed at its median value to provide a representative hydrodynamic context). We defined the primary threshold as the gain-50 threshold, namely the TSM value at which the predicted probability of tidal wetland gain reaches 0.5. This occurred at 20.4 g/m³ (see lines 771-787; Table S10).

(2) Uncertainty bounds and sensitivity testing

Uncertainty around the sediment threshold was quantified using nonparametric bootstrap resampling, yielding a 95 percent interval of 14.47 to 26.42 g/m^3 (see 780-787). This data driven interval defines the sediment threshold range used in both the discrete scenario based sensitivity analysis and the Monte Carlo uncertainty propagation (see lines 806-858, Table S5 and Fig. S4). We therefore clarify that the uncertainty bounds were not set a priori from any single published study.

(3) External benchmarking, including Kirwan et al. (2010).

We now state explicitly that published studies are used to benchmark the model derived threshold as an external plausibility check, rather than to define it. In particular, we highlight that the model derived central estimate of 20.4 g/m^3 is closely comparable to mechanistic evidence reported by Kirwan et al. (2010), which indicates an SSC threshold of about 20 mg/L for marsh survival, as well as to broader syntheses and global scale evidence (see lines 780-787, Table S10).

(4) Reporting TSM values for each site.

We agree that providing the TSM used for each site strengthens transparency and reproducibility. Accordingly, we have uploaded the site-level sediment metrics (including the long-term TSM value assigned to each restoration site) to figshare, as described in the Data Availability statement via https://figshare.com/s/90c75bdcaa4647729def.

• *L541 – referencing Kirwan et al., 2016 Overestimation of marsh vulnerability to sea level rise Nature Communications) fig 3 for example for the statement regarding acknowledgement of variation with tidal range and geomorphic setting.*

Reply: Thank you for this helpful suggestion. We agree that Kirwan et al. (2016) provides strong supporting evidence that sediment requirements and marsh persistence thresholds can vary with tidal range and geomorphic or hydrodynamic setting (see their Fig. 3). We have therefore added this reference to support our statement that sediment thresholds are not universal and may shift across tidal regimes and coastal settings (see lines 412-414). We also cite Kirwan et al. (2016) when motivating the inclusion of tidal range as a covariate in our ordinal regression analysis (see lines 594-599).

- *L544 – where are the*

Reply: Thank you for noting this. We have updated and clarified the sensitivity analysis for the global, threshold based screening, and we now describe it explicitly in the Methods (see lines 806-867). The revised sensitivity assessment has two components. First, we implemented a discrete scenario based analysis that combines three sediment thresholds with three elevation thresholds to generate nine screening scenarios, which bracket the plausible range of global estimates (see lines 806-842; Table S5). Second, we propagated threshold uncertainty using a Monte Carlo procedure, in which sediment and elevation thresholds are sampled across their uncertainty envelopes and the screening is repeated 100 times at the grid cell level (see lines 843-858; Figure S4). This Monte Carlo analysis yields spatially explicit uncertainty metrics. We report SD, CV, and the 95 percent interval width in Figure S4, and the Results show that uncertainty is generally moderate, with mean SD of 0.17 km^2 for sediment screened restoration potential and 0.09 km^2 for vegetation eligible restoration potential, corresponding to 23.9 percent and 28.6 percent of the mean cell level estimates, respectively (see lines 203-208).

- *Figure 3 – query lack of sites in Europe, eastern USA and Canada*

Reply: Thank you for this query. We agree that Fig. 3 can give the impression of apparent regional gaps in Europe, the eastern USA, and Canada. We have therefore added an explicit explanation in the Discussion that this pattern primarily reflects the structure of our loss-based screening, rather than an absence of restoration opportunities (see lines 446-465). Specifically, the candidate pool is defined by direct anthropogenic tidal wetland loss during 1999 to 2019. In Europe, the eastern USA, and Canada, mapped restoration potential can therefore appear limited mainly because eligible losses within this time window are sparse in the underlying loss datasets, which constrains the absolute area available for screening (see lines 459-465). We further clarify that the global maps should be interpreted

as a conservative, process-based screening of physically plausible recovery space. They are not intended as a comprehensive inventory of restoration pathways, and realized feasibility depends on local geomorphic context, ecosystem type, and management objectives (see lines 462-465).

In addition, we note explicitly that we updated the sediment and elevation thresholds using the revised model-informed workflow and recomputed the global screening accordingly (see lines 771-805). Fig. 3 has therefore been updated to reflect the revised thresholds and the selected screening scenario (Fig. 3).

Again, we thank all of you for these constructive comments that greatly helped improve our manuscript.

Sincerely,
Junlin REN (On behalf of all authors)

Point-by-point response

We address the reviewers’ comments below. Reviewer comments are reproduced verbatim in italics, followed by our responses in regular text. All revisions are shown in the tracked-changes manuscript, with key changes cross-referenced by line number (and by figure/table or Supplementary item where relevant).

Reviewer #1:

Reviewer #1 (Remarks to the Author):

I congratulate the authors the detailed and thoughtful revisions. The manuscript has improved substantially, particularly in the expanded geographic scope, the clarification of ecosystem boundaries, the strengthened statistical analyses, and the transparent documentation of global threshold methods. I have only a few remaining suggestions (minor) that would further enhance clarity, robustness, and reproducibility before final acceptance.

1. Lines ~ 48–58: "hydro geomorphological" → "hydro-geomorphological" (hyphenate consistently). "over 80% successfully maintained or expanded wetlands" → consider ">80% of sites maintained or expanded wetlands";

Response: Thank you for the helpful suggestions to refine the text. We have updated the manuscript accordingly. Specifically, we have consistently hyphenated "hydro-geomorphological" throughout the text. Additionally, we have adopted your suggested phrasing and changed the sentence to " over 80% maintained or expanded wetlands" to make the statement more precise (Line 51).

2. Lines ~78–80: "National and Atmospheric 2024" should be "National Oceanic and Atmospheric Administration (NOAA), 2024";

Response: We thank the reviewer for catching this error. We have corrected this reference to "National Oceanic and Atmospheric Administration (NOAA), 2024" and updated the corresponding entry in the reference list accordingly. As the manuscript now uses numerical citations, the in-text citation has also been revised to match the numbered reference format.

3. Units and symbols: Keep % yr^{-1} and g m^{-3} consistently; currently a mix of % per year and % yr^{-1};

Response: We thank the reviewer for pointing this out. All relevant units have now been consistently formatted with negative exponents (e.g., % yr^{-1} and g m^{-3}) across the main manuscript, figures, and the Supplementary Information.

4. Line 279: "wetland is expanding" → "wetlands are expanding";

Response: Corrected as suggested. The phrase has been revised to "wetlands are expanding" (Line 271). We have also thoroughly checked the manuscript for similar subject-verb agreement issues.

5. Line 437: "organgenic marshes" → "organogenic marshes";

Response: Thank you for pointing out this typo. We have corrected the spelling to "organogenic marshes". Additionally, we have conducted a comprehensive proofreading of the entire text to prevent any similar typographical errors.

6. Line ~ 799-802: Where you explain 73% (optimistic) and 104% (conservative), include one sentence that rationalizes 104% (>MHWS) as a conservative high-cover bound due to rare inundation/occasional storm/HAT events. It's there, but one consolidating sentence up front would reduce confusion;

Response: We thank the reviewer for this constructive suggestion. We fully agree that providing this ecological rationale upfront improves the clarity of our sensitivity analysis. Notably, while a final data quality check resulted in a minor refined update to the conservative threshold (from 104% to 101%), all primary findings and global spatial patterns remain unchanged. The ecological rationale you highlighted is essential for understanding this updated upper bound, and we have inserted the following consolidating sentence into the Results section:

"Notably, the conservative 101% bound represents elevations above the normal tidal frame that can still support high-marsh vegetation due to rare inundation from Highest Astronomical Tide (HAT) or occasional storm events." (Lines 192-195)

7. Use either TSM or SSC but don't mix; you use TSM consistently, which is fine—ensure units are consistently formatted as g m^{-3} (not "g m^3" or "g/m3", checking through both main text and supplementary materials);

Response: We appreciate the reviewer's positive feedback regarding our consistent use of TSM. We have conducted a thorough search across the main text, figures, and Supplementary Information to ensure that the term "SSC" is not mistakenly used. Furthermore, as suggested, we have rigorously verified that all units for TSM are correctly and consistently formatted as g m^{-3} throughout the entire manuscript and supplementary materials.

8. Database citations (ABPmer/OMREG, NOAA, ACRN) include URL + access date (ABPmer looks correct; NOAA needs fix; ACRN is cited as Purandare et al. 2024 and in the Methods—ensure consistency);

Response: We thank the reviewer for this helpful suggestion. We have standardized all three database citations (ABPmer, NOAA, and ACRN) in both the Methods and the reference list to ensure complete consistency.

Specifically, we have:

Uniform Formatting: All three entries (Refs 13-15) now consistently include the institutional author, database title, URL, and the required access date.

ACRN Consistency: To ensure uniformity, we have replaced the citation for ACRN (previously Purandare et al., 2024) with a direct database citation in both the Methods and the reference list, matching the format used for ABPmer and NOAA.

NOAA Correction: The institutional author parsing error for the NOAA database has been fixed.

9. Regarding statistical modelling

a. Beta regression treatment of 0/1 values: The site scale vegetation cover model uses a beta distribution, which excludes exact 0 or 1 values. The method does not specify how these cases were handled. Please clarify whether: you applied a transformation into (0,1) (e.g., Smithson–Verkuilen adjustment), or used a zero/one inflated beta variant. Confirming this will avoid potential misinterpretation of the model's assumptions.

Response: We thank the reviewer for this important clarification. We confirm that exact 0 and 1 values in the vegetation cover data were handled using a Smithson–Verkuilen adjustment (a linear transformation) prior to fitting the GAM with a beta error distribution. This approach compresses the data into the open (0,1) interval, making it compatible with the beta distribution's requirements. We have updated the Methods section (Lines 610-614) to explicitly describe this procedure.

b. Proportional odds assumption in the ordinal logistic model: The regional scale analysis uses an ordinal logistic regression. This model assumes proportional odds, but the manuscript does not report whether this assumption was checked. Please report the outcome of a proportional odds check (e.g., Brant test) or confirm robustness under a multinomial or partial proportional odds specification.

Response: We thank the reviewer for this helpful suggestion. In the revised manuscript, we have explicitly reported the assessment of the proportional odds assumption for the ordinal logistic regression. A Brant test was conducted, which indicated no significant deviation from the proportional odds assumption (omnibus test statistic =0.57,df = 2,P = 0.752). This confirms that the ordinal logistic specification is appropriate for our data. We have added this statistical detail to the Methods section (Lines 583-586).

c. Joint hierarchical model: The missing indicator approach used in the paper is practical but can introduce bias if missingness is not random. MCMCglmm results also depend on prior choices and convergence diagnostics. Please include a short note in SI summarizing convergence diagnostics (e.g., trace plots, effective sample sizes);

Response: Thank you for this suggestion. We have now added a short note in the Supplementary Information (**Supplementary Note 1**) summarizing the MCMC settings and convergence diagnostics for the joint hierarchical model, including the number of iterations, burn-in, thinning interval, retained posterior samples, trace plots, ESS, and lag-1 autocorrelation. In the main model, the trace plots showed adequate mixing and no obvious long-term drift, ESS values for the focal parameters ranged from 1062 to 1500, and lag-1 autocorrelation ranged from 0.015 to 0.170.

We also added three sensitivity analyses to assess whether inference depended on missing-data treatment, prior choice, or model structure. First, a complete-case analysis yielded focal effects that remained directionally consistent with the main model. Second, under an alternative prior specification (increasing the site-level covariance hyperparameter from 4 to 10), posterior summaries for the focal effects were qualitatively unchanged. Third, in an extended model including one additional hydro-geomorphological covariate per response, the focal effects for marsh cover and early-stage colonization remained positive, and the effect of TSM on wetland persistence also remained positive in direction, although with greater uncertainty (see details in Supplementary Note 1).

We now state explicitly in the SI that these analyses indicate that the main conclusions are not strongly driven by prior choice, the inclusion of missing-indicator terms, or the addition of secondary covariates, while also acknowledging that bias under non-random missingness cannot be fully excluded.

d. Sub site creek efficiency analysis (n = 18): Given the small sample size, results may be sensitive to influential observations. Suggestion: Add a one sentence note that you evaluated leverage or leave one out sensitivity, or include a partial residual plot in the SI.

Response: Thank you for this helpful suggestion. We agree that the relatively small sample size at the sub-site scale warrants an assessment of sensitivity to individual observations. We therefore conducted a leave-one-out sensitivity analysis for the drainage density model. In the full model, the relationship was significantly positive (slope = 0.0373, R^2 = 0.511, P = 0.0013). After excluding each site in turn, the fitted slope remained positive in all iterations (0.0272–0.0542), with R^2 values ranging from 0.39 to 0.65 and P values ranging from 0.0002 to 0.0093. These results indicate that the observed association was not driven by any single observation. We have added a sentence in the revised method section to report this robustness check (see Lines 660-670).

Reviewer #2:

Reviewer #2 (Remarks to the Author):

In reviewing the revisions provided to "Hydro-geomorphological drivers across scales shape the trajectory of coastal wetland restoration" - I am pleased to say that the authors have addressed all of my previous questions and concerns. The revised manuscript has significantly improved transparency , reliability and statistical robustness. It will be a significant contribution to the literature. The authors 'response to reviewers' was excellent, and very clearly outlined the changes made to the document. I have no reservations for the paper to be published in its current form.

Response: Thank you very much for your highly positive evaluation and encouraging words. We are very glad that our revisions addressed all your concerns, and we deeply appreciate your help in improving the manuscript.
